# Chemically Driven Nano‐Elastic Heterogeneities Control Fragility in Volcanic Melts

**DOI:** 10.1002/advs.202512063

**Published:** 2025-11-08

**Authors:** Michele Cassetta, Daria Szewczyk, Gabriele Giuliani, Serena Dominijanni, Francesco Vetere, Gianluca Iezzi, Francesco Radica, Dmitry Bondar, Fabrizio Di Fiore, Alessio Pontesilli, Levent Karacasulu, Nicola Daldosso, Hideyuki Mizuno, Danilo Di Genova

**Affiliations:** ^1^ Department of Engineering for Innovation Medicine University of Verona Verona I‐37134 Italy; ^2^ Department of Earth Science University of Torino Torino I‐10125 Italy; ^3^ Division of Low Temperature and Superconductivity Institute of Low Temperature and Structure Research PAS Okólna 2 Wrocław P‐50422 Poland; ^4^ Dipartimento di Scienze Università degli Studi Roma Tre Roma I‐00146 Italy; ^5^ Institute of Science, Technology and Sustainability for Ceramics (ISSMC) of the National Research Council (CNR) Via Granarolo 64 Faenza I‐48018 Italy; ^6^ Department of Physical Sciences, Earth and Environment University of Siena Siena I‐53100 Italy; ^7^ Dipartimento di Ingegneria e Geologia Università degli studi di Chieti Chieti I‐66100 Italy; ^8^ UdA‐TechLab Research Center University “G. d'Annunzio” of Chieti‐Pescara Chieti 66100 Italy; ^9^ CNR – Istituto Officina dei Materiali (IOM) c/o Department of Physics and Geology University of Perugia Perugia I‐06123 Italy; ^10^ Bayerisches Geoinstitut University of Bayreuth Universitätsstraße 30 D‐95447 Bayreuth Germany; ^11^ Istituto Nazionale di Geofisica e Vulcanologia Sezione di Roma 1 Roma I‐00143 Italy; ^12^ Department of Industrial Engineering University of Trento Trento I‐38123 Italy; ^13^ Graduate School of Arts and Sciences The University of Tokyo Tokyo 153‐8902 Japan

**Keywords:** elasticity, fragility, heterogeneities, silicate glasses, viscosity

## Abstract

Here, the nanoscale structural drivers of the mechanical behavior of volcanic glasses  are explored. The study spans a compositional series ranging from basalt to rhyolite, encompassing also technical glass‐forming melts. Using a granular‐medium framework, the vibrational and physical properties of synthetic silicate glass‐forming melts are characterized. From these, this study quantifies the correlation length (*ξ*) and microscopic free volume (*V*_c_), two parameters linked to the internal structural heterogeneity of the glass network. This study finds that both *ξ* and *V*_c_ systematically increase with silica content and show strong inverse correlations with elastic moduli and the melt fragility, highlighting how variations in medium‐range order affect the elastic and viscous response of the melt. These results provide a quantitative framework to connect compositional changes with rheological behavior, offering new perspectives on how nanoscale structural features contribute to the mechanical evolution of silicate melts.

## Graphical abstract

The study elucidates how nanoscale structural heterogeneity governs the mechanical behavior of volcanic glasses across compositions from basalt to rhyolite. Using a physicochemical framework, correlation length (*ξ*) and microscopic free volume (*V*_c_) are derived from vibrational and physical properties, revealing systematic trends with silica content that connect structural organization to the elastic and viscous response of silicate melts.

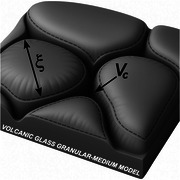

## 1 Introduction

The mechanical response of magma to deformation and cooling, particularly its transition from ductile flow to brittle failure, is fundamental to understanding volcanic eruption dynamics.^[^ ^1^ ^]^ This behavior is primarily governed by magma viscosity, which is strongly influenced by the melt's structural arrangement, especially at the medium‐range order (MRO). Even finer‐scale structural and compositional features, extending down to the nanometer scale, may play a critical role in influencing rheological behavior and, by extension, eruption style.^[^ ^2^ ^]^

At nanoscale dimensions, phenomena such as liquid‐liquid phase separation,^[^ ^3^, ^4^, ^5^ ^]^ and the nucleation of gas bubbles and crystals, ranging from tens to hundreds of nanometers,^[^ ^3^, ^6^, ^7^, ^8^, ^9^, ^10^, ^11^, ^12^ ^]^ can significantly alter the viscosity and buoyancy of magma. These changes have been suggested to influence eruptive dynamics. Moreover, insights from glass physics^[^ ^13^, ^14^, ^15^, ^16^, ^17^, ^18^, ^19^, ^20^ ^]^ suggest that structural heterogeneities at even smaller scales (few nanometers), can control deformation and viscous behavior. Such heterogeneities may govern transitions between ductile and brittle responses, often confined to localized nanodomains, “nanoductility”.^[^ ^21^ ^]^

This hierarchical structural organization, where features at progressively smaller scales affect bulk properties, can be quantified through the correlation length (*ξ*). In the context of the glassy state, *ξ* is the characteristic distance beyond which short‐range order breaks down.^[^ ^22^, ^23^, ^24^ ^]^ While glasses are inherently disordered, their structure is not random; regions of short‐range atomic order (≈5 Å) persist over specific correlation lengths, typically ranging between 5 and 20 Å.^[^ ^16^ ^]^

In volcanic melt systems, the evolution of *ξ* may be closely tied to the nucleation of nanoscale features such as gas bubbles,^[^ ^5^, ^25^ ^]^ atomic clusters,^[^ ^26^, ^27^ ^]^ and crystals.^[^ ^3^, ^19^ ^]^ One of the most effective approaches to estimate *ξ* involves analysis of the boson peak (BP), a low‐frequency vibrational anomaly that reflects an excess of vibrational modes relative to the Debye model. The position of the BP (*ω*_BP_) and the transverse sound velocity together provide a direct estimate of *ξ*, linking vibrational dynamics with medium‐range structural organization.^[^ ^28^, ^29^, ^30^ ^]^

Importantly, the vibrational density of states of a glass is also linked to the fragility (𝑚 = $\partial log\eta /\partial T_{g}/T{|}_{T=T_{g}\;}$) of its parental melt, which describes the rate of viscosity change near the glass transition temperature (*T*_g_).^[^ ^20^, ^31^, ^32^, ^33^, ^34^ ^]^ In this framework, *ξ* serves as a structural parameter that bridges dynamic processes in the supercooled liquid and mechanical behavior in the glassy state. This relationship is further complemented by the microscopic free volume (*V*_c_), which represents the atomic‐scale voids enabling structural rearrangement. Glasses with lower *V*_c_ tend to be more plastically deformable (fragile), while those with higher *V*_c_ are often rigid and brittle.^[^ ^35^, ^36^, ^37^ ^]^

In this study, we employ Brillouin, low‐frequency Raman spectroscopy, low‐temperature calorimetry, and high‐temperature viscometry to characterize the elastic and structural properties of a chemically diverse suite of volcanic melts and glasses. Specifically, we quantify how nanoscale structural parameters, correlation length (*ξ*) and free volume (*V*_c_), vary with composition, elasticity, and melt fragility. By linking the natural variability of these parameters, we aim to quantify their range in volcanic glass‐forming melts and assess how they relate to key properties such as elasticity, viscosity, and fragility, which are key in influencing eruptive dynamics.

## 2 Experimental Section

The studied samples included a large set of synthetic silicate glasses covering a broad compositional spectrum along the sub‐alkaline basalt–rhyolite join. Specifically, six glass compositions were selected and previously synthesized and characterized by Vetere et al.^[^ ^38^ ^]^ In their study, two natural rock samples, a basalt from Iceland (B_100_) and a rhyolite from Lipari Island, Italy (R_100_), were first melted to produce homogeneous end‐member glasses. These end‐members were subsequently mixed in different weight‐proportions (80:20, 60:40, 40:60, and 20:80) and re‐melted to obtain four intermediate compositions (B_80_R_20_, B_60_R_40_, B_40_R_60_, and B_20_R_80_). In order to have sufficient material for viscosity measurements and to compare the materials, two glasses having the same compositions have been re‐synthesized as R_100_ and B_80_R_20_ and named R_100‐n_ and B_80‐n,_ respectively (see composition in **Table** **1**). Notably, this re‐synthesis resulted in a different iron oxidation state for the rhyolitic sample, allowing for a direct assessment of its influence on the glass's physical properties, as detailed in the Discussion section.

**Table 1:** EPMA analyses in wt.% of glasses from the sub‐alkaline basalt–rhyolite join from ref. [38] Standard deviations are within ± 0.4 for SiO_2_, Al_2_O_3_, FeO, and CaO, while within ± 0.1 for all the other presented oxides.

	B_100_	B_80_R_20_	B_60_R_40_	B_40_R_60_	B_20_R_80_	R_100_	B_80‐n_	R_100‐n_	NVP
SiO_2_	48.02	53.01	57.97	62.73	67.91	73.97	52.98	72.34	61.48
TiO_2_	0.98	0.80	0.65	0.46	0.29	0.12	0.81	0.13	0.36
Al_2_O_3_	15.59	14.99	14.62	14.05	13.59	13.48	15.13	13.48	8.95
FeO_(t)_	10.23	8.54	6.95	5.42	3.69	2.06	8.29	2.18	‐
MnO	0.18	0.15	0.13	0.12	0.11	0.08	0.18	0.11	‐
MgO	9.42	7.58	5.81	4.01	2.18	0.44	6.92	0.45	14.12
CaO	13.20	10.79	8.46	6.07	3.63	1.36	10.89	1.36	6.81
Na_2_O	1.79	2.18	2.59	2.95	3.29	3.75	2.11	3.60	8.85
K_2_O	0.04	1.02	1.99	3.02	3.99	4.89	0.95	4.65	0.21
P_2_O_5_	0.06	0.02	0.04	0.02	0.02	0.03	0.03	0.03	‐
Tot.	99.90	99.53	99.64	99.26	99.12	100.52	98.28	98.33	100.78
Fe^2+^/Fe_tot._	0.39	0.45	0.43	0.41	0.42	0.34	‐	‐	‐
NBO/T	0.88	0.68	0.50	0.34	0.19	0.06	0.69	0.06	0.88
*ρ* [g cm^−3^]	2.804	2.648	2.599	2.460	2.366	2.238	2.725	2.355	2.587
*C*_g_	0.516	0.496	0.495	0.477	0.467	0.449	0.507	0.471	0.508

Additionally, eight calc‐alkaline rhyolitic glasses (Rh series) were examined, one andesitic glass from Montserrat (MSA), and one basaltic glass from Stromboli (Str), previously investigated in.^[^ ^2^, ^33^ ^]^ To further broaden the compositional and structural range, data were included from extremely depolymerized peridotitic glasses reported in,^[^ ^39^ ^]^ as well as highly Ca‐ and Mg‐enriched phonotephritic glasses studied in.^[^ ^40^ ^]^ Finally, to enable comparison with terrestrial silicate melts, an extraterrestrial glass was analyzed and synthesized as an analog for Mercury's Northern Volcanic Plains (NVP) lava compositions.^[^ ^41^ ^]^

To contextualize the volcanic glass‐forming melts, a comparison set of technical glasses were assembled with published viscosity and spectroscopic data, including mineral‐analog glasses, (diopside, Di, and anorthite An^[^ ^33^ ^]^); standard soda‐lime (DGG‐1,^[^ ^33^ ^]^ SRM‐710^[^ ^42^, ^43^ ^]^); a standard borosilicate glass of Pyrex composition;^[^ ^44^, ^45^ ^]^ a binary set of soda‐silica *x*Na_2_O − (1 − *x*)SiO_2_;^[^ ^34^ ^]^ and a potassa‐disilicate glass.^[^ ^46^, ^47^, ^48^, ^49^ ^]^

The chemical compositions, iron speciation (Fe^2+^/Fe_tot._), and density (*ρ*) of all samples are summarized in Tables 1 and 2. Literature values for chemical composition, sound velocities; and melt fragility are reported in Tables SI‐1–SI‐4 (Supporting Information).

**Table 2:** Longitudinal and transverse sound velocities (*v*_l_ and *v*_t_), calculated elastic moduli (*G*, *K*, *M*, and *E*), Poisson's ratio (*σ*), and glass transition temperatures (*T*_g_) for the B_100_–R_100_ glass series.

	B_100_	B_80_R_20_	B_60_R_40_	B_40_R_60_	B_20_R_80_	R_100_	B_80‐n_	R_100‐n_	NVP
*v*_l_ [m s^−1^]	6502 (12)	6315 (8)	6188 (16)	6041 (9)	5888 (10)	5744 (9)	6341 (17)	5775 (10)	6209 (8)
*v*_t_ [m s^−1^]	3629 (8)	3592 (10)	3587 (10)	3574 (8)	3550 (8)	3535 (10)	3609 (9)	3547 (9)	3623 (6)
*G* [GPa]	36.9 (2)	34.2 (2)	33.4 (2)	31.4 (2)	29.8 (3)	27.9 (4)	35.5 (2)	29.6 (2)	33.9 (1)
*K* [GPa]	69.3 (6)	60 (6)	54.9 (6)	47.9 (4)	42.3 (8)	36.6 (1.1)	62.2 (8)	39.0 (5)	54.5 (4)
*M* [GPa]	118.6 (5)	106 (5)	99.5 (6)	89.8 (4)	82.0 (7)	73.8 (1.0)	109.6 (7)	78.5 (4)	99.7 (3)
*E* [GPa]	94.1 (6)	86 (7)	83.4 (6)	77.4 (5)	72.4 (8)	66.9 (1.2)	89.5 (6)	70.9 (6)	84.3 (4)
*σ*	0.27 (0)	0.26 (0)	0.25 (0)	0.23 (0)	0.21 (1)	0.20 (2)	0.26 (0)	0.20 (1)	0.24 (0)
*T*_g_ [°C]	698.2	699.7	706.0	713.5	730.8	752.4	672.4*	771.5*	661.8*

The atomic packing density (*C*_g_) is calculated following:

(1) $$C_{g}=\rho \;\frac{\sum f_{i}V_{i}}{\sum f_{i}M_{i}}$$

where, for the 𝑖‐th oxide component written as A*_x_* B*_y_*: *V_i_* = 4/3π*N_A_* ($xr_{A}^{3}+yr_{B}^{3}$), where *ρ* is the density, *N_A_* is the Avogadro's number, *r*_A_ and *r*_B_ are the ionic radii of ions *A* and *B*, *f_i_* is the molar fraction, and *M_i_* is the molar mass of the component *i*. This parameter was important for assessing glass elasticity, as it correlateed with the magnitude of the elastic moduli.^[^ ^50^ ^]^

### 2.1 He‐Pycnometry

Glass density of B_100‐n_, B_80‐n,_ and NVP was determined using an Anton Paar Ultra‐pyc5000 helium pycnometer at the University of Trento. Each value in Table 1 corresponds to the mean of 10 measurements, carried out at 20 °C under pulse mode (10 pulses) with a pressure setting of 10 psi.

### 2.2 Electron Probe Microanalysis

Major element compositions of the glasses were measured using a JEOL JXA‐8200 electron probe microanalyzer (EPMA) at the Bayerisches Geoinstitut (BGI, Bayreuth), equipped with five wavelength‐dispersive spectrometers. Prior to analysis, samples were carbon‐coated to a thickness of ≈12 nm. Measurements were performed with a defocused electron beam of 10 µm diameter, using an accelerating voltage of 15 kV, a beam current of 5 nA, and counting times of 20 s per element. Oxygen content was calculated stoichiometrically, assuming all iron is present as FeO. Calibration standards included wollastonite (Si, Ca), hematite (Fe), periclase (Mg), spinel (Al), albite (Na), orthoclase (K), and manganese titanate (Ti, Mn).

### 2.3 Low‐Frequency Raman Scattering

Low‐frequency Raman scattering (LOFIRS) spectra were acquired under crossed‐polarization (HV) conditions using two different spectrometers, depending on the sample type:

i) For sample B_100_‐R_100_, B_80‐n,_ and NVP glasses, measurements were performed using a Horiba Jobin‐Yvon T64000 triple‐monochromator spectrometer operating in double subtractive/single mode (at the Raman Spectroscopy Lab of the Center for Technological Platforms at the University of Verona, Italy). Excitation was provided by a mixed Ar–Kr ion laser (Spectra Physics Satellite 2018 RM) at 514.5 nm. The laser beam was focused to a ≈2 µm spot using a 50X objective (N.A. = 0.75), with a power of ≈10 mW at the sample surface. The scattered light was filtered using three holographic gratings (1800 lines mm^−1^) and detected with a liquid‐nitrogen‐cooled CCD (1024 × 256 pixels). All samples were polished prior to analysis and optically inspected before and after measurement to ensure the absence of laser‐induced damage at the micron scale. Stokes‐shifted spectra were collected over two overlapping spectral windows (10–650, and 650–1300 cm^−1^) to cover the full vibrational range. Low‐wavenumber spectra were corrected by subtracting the rotational Raman signal of air below 180 cm^−1^.
ii) For R_100‐n_, Ca‐ Mg‐doped phonotephritic and peridotitic glasses, spectra were obtained using a WITec Alpha300R Raman spectrometer equipped with a RayShield ultra‐low‐frequency filter and a crossed‐polarized optical configuration at the GLASS (Gateway Laboratory of Amorphous and Structured Solids and Melts), CNR–ISSMC in Rome (Italy). The system included a lens‐based imaging spectrometer (300 mm focal length) with a motorized triple grating turret (600 and 1800 lines/mm, blazed at 500 nm). Detection was achieved with a back‐illuminated CCD (1024 × 127 pixels, 26 × 26 µm pixel size), Peltier‐cooled to −50 °C. A laser power of 5 mW was used at the sample surface. To minimize interference from weak luminescence, a linear baseline subtraction was applied, preserving the integrity of the low‐frequency spectral region (10–200 cm^−1^).

### 2.4 Brillouin Spectroscopy

Brillouin frequency shifts were measured at BGI on B_100_–R_100_ and NVP glasses using a six‐pass Fabry–Pérot interferometer coupled with a single‐pixel photon counter. The interferometer was equipped with double‐sided polished optical glass plates (50 µm thick). Measurements were conducted at ambient temperature in platelet geometry using a 532 nm Nd:YVO_4_ laser operating at 50 mW as the excitation source. A symmetric forward‐scattering geometry was employed with a calibrated scattering angle of 79.8°, based on reference measurements in silica glass.

Brillouin frequency shifts (Δ*ω*) were converted to longitudinal (𝑣𝑙) and transverse (𝑣𝑡) sound velocities using the relation:

(2) $$v=\frac{\Delta \omega \lambda }{2\;\sin\;(\theta /2)}$$

where 𝜆 is the laser wavelength in air, and 𝜃 is the scattering angle. For each sample, 8 to 9 spectra were collected at varying rotation angles (−180° to +180°) to reduce uncertainty and improve measurement reliability.

### 2.5 Low Temperature Heat Capacity

Low‐temperature heat capacity (LTHC) measurements were conducted on B_100_–R_100_ glass samples using the Heat Capacity Option of a Quantum Design Physical Property Measurement System (PPMS®). Measurements were performed over the temperature range of 1.8–50 K using the standard thermal relaxation method, which accounts for non‐ideal thermal coupling between the sample and the measurement platform.

Prior to each final measurement, an additional experimental run was carried out to quantify and subtract the contribution of the mounting grease to the total measured heat capacity. Small fragments of glass (ranging from 6.1 to 10.8 mg) were used to ensure optimal thermal contact and compatibility with the measurement platform.

The heat capacity of the glass sample was determined using the following system of differential equations:

(3) $$C_{\mathrm{platform}}\frac{dT_{p}}{dt}=P\left(t\right)\;-\;K_{w}\left[T_{p}\left(t\right)\;-\;T_{b}\right]+K_{g}\left[T_{p}\left(t\right)\;-\;T_{s}\left(t\right)\right]$$

(4) $$C_{\mathrm{sample}}\frac{dT_{s}}{dt}=-K_{g}\left[T_{p}\left(t\right)-T_{s}\left(t\right)\right]$$

where *C*_platform_ is the heat capacity of the measuring platform including the grease, *C*_sample_ is the heat capacity of the sample, *P*(*t*) is the power applied by the heater to the sample,  *K*_w_ is the thermal conductance of the supporting wires, and *K*_g_ is the thermal conductance of the grease layer between the sample and platform. *T*_p_(*t*) and, *T*_b_ and *T*_s_(*t*) are the temperatures of the platform, thermal bath, and sample, respectively. Further details on the experimental setup and thermal modeling can be found in.^[^ ^51^ ^]^

### 2.6 Differential Scanning Calorimetry

Differential scanning calorimetry (DSC) measurements were conducted on B_100_–R_100_ glass samples using a Netzsch 404 F3 Pegasus C‐DSC system at the Experimental Volcanology and Petrology Laboratory (EVPLab) at the University of Roma Tre (Italy). Approximately 15 ± 5 mg of each glass was placed in a platinum crucible and analyzed under a constant nitrogen (N_2_ 5.0) flow of 30 mL min^−1^. Heating rates ranged from 10 to 20 K·min^−1^. Calibration of the DSC was performed using standard reference metals (indium, tin, bismuth, zinc, aluminum, silver, and gold), based on their known melting points and enthalpies of fusion.

To remove thermal history effects, samples were first heated above the glass transition interval at 20 K min^−1^ and subsequently cooled to 100 °C at the same or a slightly lower rate (10 or 20 K·min^−1^). The measurements followed the rate‐matching method, in which the second heating (upscan) was performed at the same rate as the preceding cooling segment (downscan), allowing accurate detection of the glass transition and related thermal properties.

Following, two different heating–cooling rates, 10 and 20  K min^−1^, were applied on sample NVP to determine the viscosity values using the shift‐factor method described in.^[^ ^52^ ^]^

(5) $$\log\;\eta_{l}\;\left(T_{\mathrm{onset},\mathrm{peak},\mathrm{endset}}\right)=K_{\mathrm{onset},\mathrm{peak},\mathrm{endset}}-\;\log\;\left(\left|q_{c,h}\right|\right)$$

Here, *q*_c,h_ stands for heating‐cooling rates at which the *T*_onset_ *_,_ T*_peak_ *_,_ T*_endset_ were measured, while *K*_onset_ = 11.20, *K*_peak_ = 9.84, and *K*_endset_ = 9.21 are the parallel shift factors.^[^ ^52^ ^]^ This approach enabled us to estimate viscosity at different structural relaxation states in the glass transition region.

### 2.7 Concentric Cylinder and Micropenetration Viscometry

High‐temperature viscosity measurements were performed at EVPLab on R_100‐n_ and B_80‐n_. A concentric cylinder (CC) setup equipped with a Rheotronic II Rotational Viscometer (Theta Instruments) and an Anton Paar Rheolab QC viscometer head (torque capacity: 75 mNm) was used in this study. The measurement assembly included a Fe‐presaturated Pt_80_Rh_20_ crucible (62 mm height, 32 mm inner diameter, and 1.5 mm wall thickness) and a Fe‐presaturated Pt_80_Rh_20_ spindle (3.2 mm diameter and 42 mm length).

To ensure melt homogeneity, samples were stirred at a shear rate of 10 s^−1^ for 3 h prior to viscosity measurements. Instrument calibration was performed using the NIST 717a viscosity standard material, achieving a precision of ±0.06 log units; details of the calibration protocol are reported in.^[^ ^53^, ^54^ ^]^ Temperatures were measured with an S‐type thermocouple, factory‐calibrated to an accuracy of ±2 °C. Viscosity measurements were conducted at ambient pressure and air oxygen fugacity.

Viscosity measurements were carried out for over a temperature range of 1525–1218 °C, corresponding to fully superliquidus conditions. The temperature was decreased in increments of 25–50 °C (see Table SI‐2, Supporting Information). At each step, the melt was held for 30–60 min to ensure thermal and mechanical steady‐state before recording viscosity values.

Plane‐parallel glass chips, each 3 mm thick, were used for micropenetration viscometry (MP) measurements. A sapphire sphere with a radius of 0.75 mm was employed to measure the penetration rate using a thermomechanical analyzer (TMA/SDTA 2+, Mettler Toledo) at GLASS. The TMA was calibrated using viscosity measurements of the standard glass DGG‐1. The obtained values were consistent with the certified viscosity data^[^ ^55^ ^]^ within a deviation of ± 0.1 log units. Additionally, furnace temperature calibration was performed using the melting points of standard reference materials (indium, zinc, aluminum, silver, and gold), and the thermal lag was determined based on temperature variations recorded via the SDTA signal.

## 3 Results and Discussion

### 3.1 Elastic Properties and Glass Transition Temperature

Bulk densities (ρ) were determined by measuring the mass and dimensions of highly isotropic glass prisms to calculate their volumes (Table SI‐3, Supporting Information). A systematic decrease in density was observed across the compositional join, from basalt (B_100_) to rhyolite (R_100_).

Longitudinal (*v*_l_) and transverse (*v*_t_) sound velocities, reported in **Table** **2**, decrease from 6502 and 3629 m s^−1^ in B_100_ to 5744 and 3535 m s^−1^ in R_100_, respectively. The largest variation is observed in the longitudinal wave velocity, indicating its greater sensitivity to compositional changes compared to the transverse component. From these measurements, we calculated the longitudinal modulus (*M*), shear modulus (*G*), bulk modulus (*K*), Young's modulus (*E*), and Poisson's ratio (*σ*), following standard formulations.^[^ ^56^ ^]^

The elastic properties show a clear decreasing trend with increasing SiO_2_ content. Specifically, *M* decreases by 38%, *G* by 24%, *E* by 29%, and *σ* by 26% from B_100_ to R_100_. The most significantly affected parameter is the bulk modulus (*K*), which decreases by 47% across the compositional join. This trend reflects the progressive polymerization and structural weakening of the glass network as silica content increases.

Overall, lower degrees of polymerization correspond to higher elastic moduli and sound velocities, consistent with previous observations in both haplo‐volcanic glass systems (i.e., basalt to granite:^[^ ^57^, ^58^ ^]^) and mineral glass analogs (i.e., diopside to anorthite:^[^ ^56^ ^]^). Among the elastic parameters, Poisson's ratio (σ) is particularly informative, as it reflects the material's resistance to shape change relative to volume change. Low σ values are typical of shear‐resistant, compressible materials, whereas higher values indicate reduced compressibility.

In our dataset, *σ* scales with the atomic packing density (*C*_g_). In alkali silicates, increasing alkali content raises *C*_g_ and, correspondingly, σ.^[^ ^46^ ^]^ Alkali–aluminosilicate glasses show a less monotonic response: although adding Al generally lowers *C*_g_, σ can increase when Al adopts sixfold (octahedral) coordination. As a result, σ often rises at low Al contents, decreases as Al_2_O_3_/Na_2_O approaches 1, and then increases again with further Al enrichment.^[^ ^59^ ^]^ A comparable trend occurs in (Mg,Ca)‐aluminosilicate glass systems,^[^ ^56^ ^]^ where substituting the smaller Mg cation for Ca or Si increases packing; however, the associated disruption of the silica network weakens Si–O–Si linkages, moderating *σ* despite the higher structural compactness.

These structural adjustments reflect increasing chemical complexity and denser local atomic environments, which enhance the bond strength at the sub‐nanometer scale. Consequently, glasses with higher *C*_g_ often exhibit increased *E* and *σ*, particularly within compositionally related glass systems, i.e. within families of glasses sharing similar chemistry or dominated by a common network modifier or silica content.^[^ ^50^ ^]^ However, some discrepancies in physical properties remain between original and the replicants samples, most notably in R_100n_. Given the very similar chemical composition, this mismatch may stem from differences in the oxidation state of iron, which are explored in more detail below. The glass transition temperature (*T*_g_) increases systematically from basaltic (B_100_) to rhyolitic (R_100_) compositions, rising by more than 50 °C across the series. This trend is opposite to that of the elastic properties (*v*_l_ *, v*_t_ *, G, K, M, and E*), which all decrease with increasing silica content. While elastic moduli and sound velocities reflect the weakening of interatomic stiffness and packing efficiency in more polymerized networks, *T*_g_ tracks kinetic hindrance to structural relaxation: as polymerization increases (lower NBO/T), viscosity grows, and a higher temperature is required to reach the glass transition criterion *η*(*T*_g_) = 10^12^ Pa s. Thus, the inverse correlation between *T*_g_ and elastic stiffness underscores the distinction between equilibrium elastic response and kinetic arrest during cooling in volcanic glass‐forming melts.

The apparent contrast, where basalts exhibit higher solidus and liquidus temperatures, yet rhyolites display higher *T*_g_, further reflects the distinction between thermodynamic phase equilibria, governed by compositional controls on crystal melting relations, and the relaxation‐kinetic nature of *T*_g_, which rises with increasing melt polymerization and viscosity as silica content increases.

### 3.2 Vibrational Density of States

**Figure 1:**
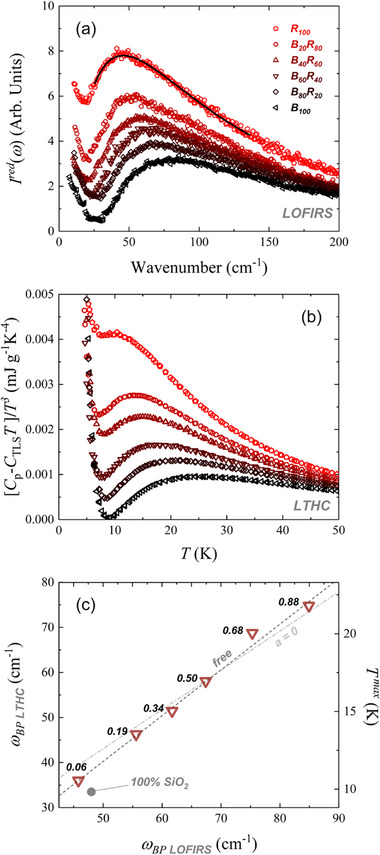
a) LOFIRS spectra for the B_100_‐R_100_ series, with the black line indicating the log‐normal fits used to determine the boson peak (BP) position (*ω*_BP_) (see Equation 6). b) Plot of LTHC data expressed as [*C*_p_‐*C_TLS_T*]/*T* ^3^, highlighting the BP anomaly. c) Correlation between *ω*_BP_ values obtained from LOFIRS and LTHC. The right vertical axis indicates the corresponding *T^max^* values (in *K*). Gray lines show linear regressions: dashed line corresponds to the fit with free intercept (*ω*_BP,LTHC_ = −10.38(±2.28) + 1.015(±0.03)*ω*_BP,Raman_); dash‐dotted line represents the fit with intercept fixed to zero (*ω*_BP,LTHC_ = 0.864(±0.01)*ω*_BP,Raman_). Labels indicate the non‐bridging oxygen per tetrahedron (NBO/T) values for each sample.

The reduced LOFIRS spectra of the B_100_‐R_100_ glass series (**Figure** **1**a) exhibit a characteristic boson peak (BP) below ≈200 cm^−1^, an excess of vibrational modes over the Debye model prediction, *g*(*ω*)/*ω* ^2^.^[^ ^29^, ^30^ ^]^ This low‐frequency vibrational anomaly is also observable in LTHC (*C*_p_) data below ≈50 K (Figure 1b), providing a robust proxy for assessing MRO in glasses based on pure and thermally‐dependent vibrations.^[^ ^60^, ^61^ ^]^

To isolate the BP from overlapping spectral features, particularly the intense polarized band associated with tetrahedral ring vibrations (R band, between 460 and 600 cm^−1^), we used cross‐polarized (HV) Raman configuration.^[^ ^62^, ^63^, ^64^ ^]^ The Raman spectra were converted to reduced intensity *I^red^* (*ω*), using the^[^ ^65^ ^]^ relation:

(6) $$I^{red}(\omega )=\;\frac{I^{obs}}{\omega \;\left[n(\omega ,T)+1\right]}=C(\omega )\frac{g(\omega )}{\omega^{2}}$$

where, *n*(*ω*,*T*) is the Bose‐Einstein distribution, *n*(*ω*,*T*)  =  [exp(ℏ*ω*/*k_B_T*)‐1]^−1^, *C*(*ω*) is the light–vibration coupling function, and ℏ and *k_B_* are the reduced Planck's and Boltzmann constants, respectively. Above the quasi‐elastic scattering, from 10 to 120 cm^−1^ the dependency of *C*(*ω*) ∝ *ω* ^α^ (α as system‐dependent exponent) provides a frequency range of 0.5*ω*_BP_–1.5*ω*_BP_.

Following, the Raman BP position *ω*_BP_ (Tab. 3) was determined by fitting the LOFIRS spectra to a log‐normal function^[^ ^66^ ^]^ in the form of *I*(ω) ∝ exp{− [ln(*ω*/*ω*_BP_)]^2^/2Γ^2^}, where Γ is the BP width and *ω*_BP_ its position in cm^−1^.

**Table 3:** Debye frequency (*ω*_D_), boson peak position (*ω*_BP_), maximum heat capacity temperature (*T* ^*max*^), and correlation length (*ξ*) for the B_100_–R_100_ glasses.

	B_100_	B_80_R_20_	B_60_R_40_	B_40_R_60_	B_20_R_80_	R_100_	B_80‐n_	R_100‐n_	NVP
*ω*_D_ [THz]	10.6	10.4	10.3	10.1	9.9	9.7	10.5	9.9	10.4
*ω*_D_ [cm^−1^]	355.2	345.8	343.9	337.1	331.3	324.4	350.8	331.2	346.9
*T^max^* [K]	21.8	20.1	16.9	15.0	13.5	10.5	‐	‐	‐
*ω*_BP,LOFIRS_ [cm^−1^]	84.9	75.3	67.4	61.7	55.6	45.8	76.3	49.2	78.3
*ω*_BP,VDoS_ [cm^−1^]	75.8	66.1	58.1	52.3	46.1	36.1	67.1	39.6	69.1
*ξ* [nm]	1.60	1.81	2.06	2.28	2.57	3.26	1.79	2.99	1.75

As shown in Figure 1a, the BP shifts systematically to higher frequencies and decreases in intensity as glass composition shifts from rhyolitic (R_100_) to basaltic (B_100_), indicating increased glass density and reduced polymerization.

LTHC measurements (Figure 1b) display a notable upturn below ≈50 K, consistent with iron‐bearing silicate glasses.^[^ ^27^ ^]^ The data were modeled using a combination of two‐level systems (TLS), Debye, and soft‐potential model (SPM) contributions:^[^ ^61^ ^]^:

(7) $$C_{p}=C_{\mathrm{TLS}}T+C_{\mathrm{Debye}}T^{\;3}+C_{\mathrm{SPM}}T^{\;5}$$

The BP features obtained from heat capacity data exhibit similar trends to those observed in Raman spectra, with both *ω*_BP_ and the heat capacity maximum (*T^max^*) shifting to higher frequencies and temperatures, respectively, with increasing melt depolymerization and density.

Unlike optical spectroscopies, LTHC is not limited by vibrational mode selection rules, and thus captures the full vibrational density of states. The Einstein model allows direct correlation between the maximum in *C_p_*/*T* ^3^ and the BP energy:

(8) $$T^{max}=\frac{1}{39763.211}\left(\frac{\omega_{BP}}{k_{B}}\right)$$

Figure 1c illustrates the linear correlation between *ω*_BP_ from Raman data and *T^max^* from heat capacity, yielding a slope of ≈1.01 and intercept of ≈10.38, suggesting similar coupling behavior to that of vitreous SiO_2_. Supporting this, Figure SI‐1 (Supporting Information) shows the correlation between *ω*_BP_ **/** *k_B_* (Raman‐derived) and *T^max^*, with a slope of ≈5.69, closely matching the value (≈5) found by Carini et al. 2016^[^ ^67^ ^]^ for a wide range of glass compositions and densification states.

These observations support the view that the BP arises from localized soft vibrational modes interacting with delocalized phonons, forming spatially correlated patterns at the nanometer scale, features whose size and energy depend on melt composition and structural complexity.^[^ ^23^, ^68^, ^69^, ^70^ ^]^

**Figure 2:**
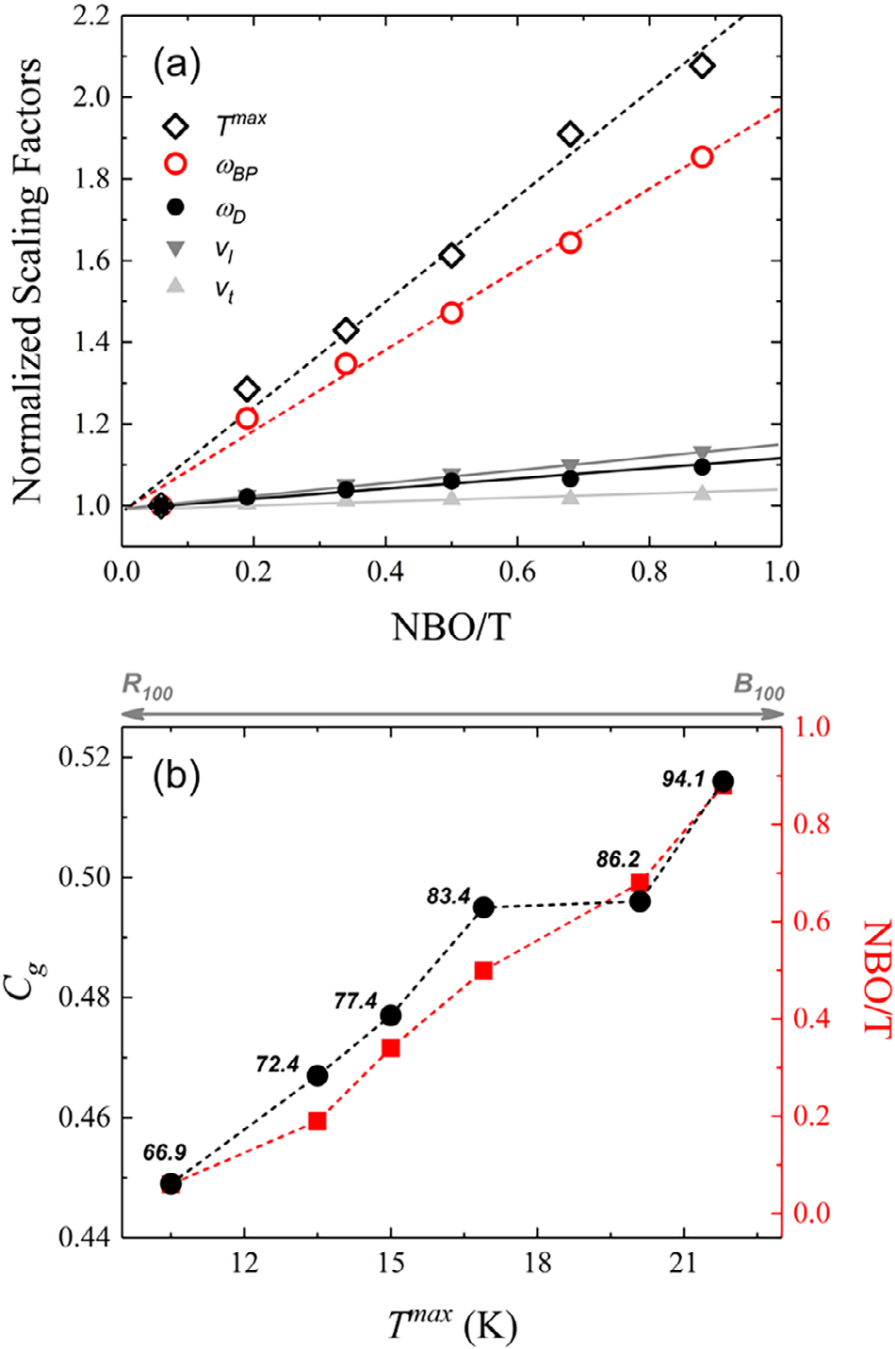
a) Boson peak (BP) position derived from reduced Raman intensity (*I^red^*, black diamonds) and the corresponding *T^max^* values from low‐temperature heat capacity (LTHC) measurements (red circles), plotted as a function of the degree of depolymerization (NBO/T). Solid symbols represent Brillouin light scattering (BLS)‐derived sound velocities and the corresponding Debye frequency (*ω*_D_). b) Relationship between atomic packing density (*C*_g_) and the Raman‐derived BP energy normalized by the Boltzmann constant (*ω*_BP_/*k*_B_), shown alongside variations in NBO/T. Labels indicate the Young's modulus (*E*) for each composition, highlighting the connection between structural compactness, vibrational properties, and elastic behavior.

To assess the relationship between ω_BP_ and the elasticity of the glass network, we compared the BP to the Debye frequency (*ω*_D_ = 1$8\pi^{2}n[(1/v_{l}^{3})+(2/v_{t}^{3}{)]}^{-1}$, with *n* number density) to the network polymerization (NBO/T, non‐bridging oxygen per tetrahedron^[^ ^71^ ^]^). **Figure** **2**a shows that the shift in *ω*_BP_, derived from both Raman and LTHC measurements, correlates more strongly with NBO/T than with *ω*_D_, suggesting a direct link between vibrational excess and glass structure (**Table** **3**).

Finally, Figure 2b highlights an inverse relationship between the atomic packing density (*C*_g_) and the BP energy. This trend mirrors the variation in NBO/T that is accompanied by a notable increase in Young's modulus (*E*), emphasizing how volumetric nanoscale packing influences both vibrational and mechanical properties in volcanic systems.

### 3.3 Nanoscale Heterogeneities

If the BP reflects the intrinsic heterogeneity of glasses, then the correlation length (*ξ*), the characteristic distance over which short‐range structural order is preserved, can be derived as a direct proxy for nanoscale disorder.^[^ ^13^, ^16^, ^18^, ^22^, ^23^ ^]^ This length scale can be estimated using the relation:

(9) $$\xi =\frac{v_{t}}{\omega_{\mathrm{BP}}}$$

where *v_t_* is the transverse sound velocity and *ω*_BP_ is the boson peak position (Equation 8). This approach is justified since the BP is primarily associated with transverse vibrational modes,^[^ ^28^ ^]^ as demonstrated by both depolarization ratios in LOFIRS spectra^[^ ^72^ ^]^ and numerical simulations.^[^ ^73^, ^74^ ^]^

**Figure 3:**
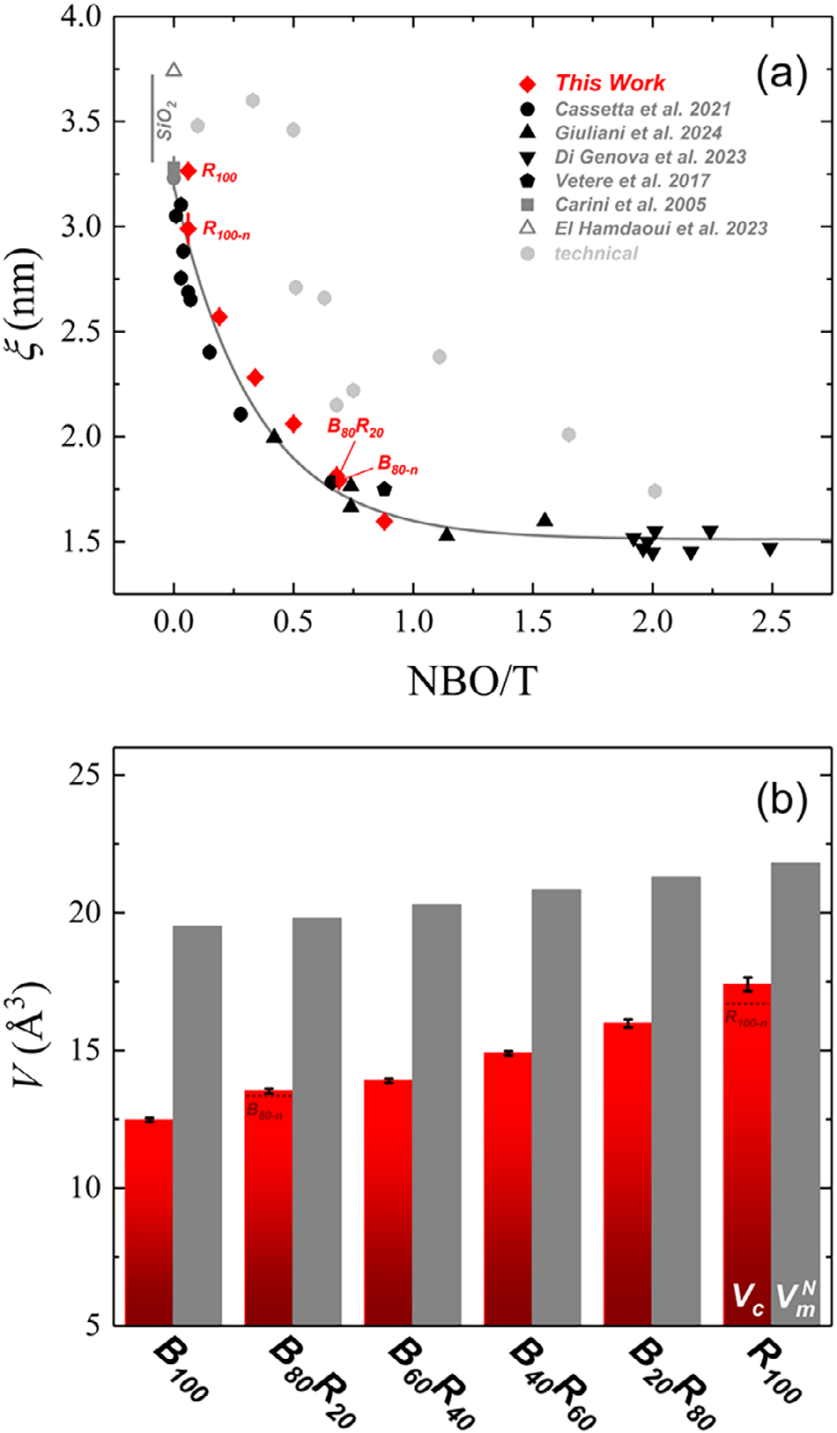
a) Correlation length (*ξ*, Equation 9) as a function of the non‐bridging oxygen per tetrahedron (NBO/T) for subalkaline volcanic glasses derived from low‐temperature heat capacity (LTHC) measurements (red diamonds). Additional compositions from dataset are shown in black, including glassy SiO_2_ (gray). b) Variation of normalized molar volume ($V_{m}^{N}=V_{m}\;/N$) and microscopic free volume (*V_c_*, Equation 11) as a function of NBO/T. The trends highlight the influence of melt depolymerization on nanoscale packing and structural heterogeneity.

**Figure** **3**a shows a clear inverse correlation between *ξ* and NBO/T, with *ξ* increasing by ≈44% from ≈1.60 nm in basalt to ≈3.26 nm in rhyolite. This suggests a transition toward greater elastic heterogeneity and a more defined separation between rigid and soft domains across the compositional series. When extended to the entire dataset (Figure 3a; Table SI‐4, Supporting Information), this trend is well described by an exponential decay model:

(10) $$\xi =\xi_{0}+Ae^{-(\mathrm{NBO}/T)/B}$$

here, *ξ*_0_ represents the limiting value of ξ at high depolymerization (i.e., ultrabasic melts), 𝐴 is the excess correlation length at NBO/T = 0 (i.e., for SiO_2_), and 𝐵 is a decay constant that reflects how quickly the correlation length shortens with increasing NBO/T.

To probe the structural implications of this trend, we compared the correlation length (*ξ*) with the system's free volume. Specifically, we estimated the microscopic activation volume *V*_c_, (parent‐liquid derived), which quantifies the dilational volume associated with viscous flow and reflects the structure of the liquid from which the glass was quenched.^[^ ^75^ ^]^

(11) $$\eta \;\left(T\right)=\eta_{\infty }\exp\left[\frac{G\left(T\right)V_{c}}{k_{B}T}\right]$$

where *η*(*T*) is viscosity at temperature *T* (in K), *η*_∞_ = 10^−2.93^ Pa s^[^ ^76^, ^77^ ^]^ is the high‐temperature viscosity limit, *G*(*T*) is the shear modulus (assumed constant below *T*_g_), *k*_B_ is Boltzmann's constant, and *V*_c_ is the microscopic activation volume. This model assumes that viscosity reflects the structure “frozen in” at the fictive temperature (*T*_f_), with negligible structural relaxation during measurements due to short timescales and consistent cooling rates. Indeed, *G*(*T*) variations during heating below *T*_g_, influences *V*_c_ only by the 3% in silicate glasses.^[^ ^78^ ^]^

We also calculated the normalized molecular volume $V_{m}^{N}=V_{m}\;/N$, the volume occupied by a single ‘molecule’ of the glass network, which we use as a simple gauge of how tightly the structure is packed^[^ ^79^ ^]^ As shown in Figure 3b and Table SI‐4 (Supporting Information), both *V*_c_ (the local volume that must dilate for the melt to flow) and $V_{m}^{N}$ increase from basalt to rhyolite, consistent with a more expanded silica‐rich network. However, the gap between these two volumes decreases along the series, indicating that rhyolite is more structurally uniform at the nanoscale than basalt.

In practical terms, rhyolitic glasses are more open but more evenly packed, which helps explain their higher viscosity and slower, more constrained relaxation. In contrast, basaltic glasses, which have smaller volume, are more prone to rapid structural relaxation and deformation, potentially influencing their rheological behavior and how magma flows during volcanic eruptions. Indeed, the free volume scales with the activation volume for viscous flow, and the energy required to form a tiny void tracks the activation energy for flow.^[^ ^35^, ^36^, ^37^ ^]^ Therefore, the interplay between structural relaxation, spatial distribution of elastic heterogeneity (*ξ*), and free volume plays a central role in controlling the mechanical behavior of volcanic melts.

### 3.4 Heterogeneities and Melt Fragility

The relationship between melt fragility 𝑚 and structural heterogeneity is critical for understanding both glass formation and volcanic processes, as fragility controls how rapidly viscosity changes near the glass transition temperature (*T*_g_). In general, as 𝑚 increases, the correlation length (*ξ*) between molecular domains decreases, reflecting reduced structural coherence. Similar patterns have been observed in molecular glass formers and colloidal systems, where dynamic slowdowns during glass formation are driven by growing correlated regions.^[^ ^80^ ^]^

Additionally, materials with a higher density of soft elastic modes often exhibit larger‐scale shear fluctuations (higher *ξ*), leading to enhanced mechanical strength and reduced Poisson's ratio.^[^ ^16^ ^]^ The melt fragility inversely correlates with medium‐range structural features in glasses, particularly the abundance and size of silica rings.^[^ ^20^ ^]^ Glass‐forming melts with larger, more stable ring structures resist deformation over broader temperature intervals and thus exhibit lower fragility. Consequently, strong (low‐𝑚) systems require a larger temperature excursion to achieve a given change in viscosity, whereas fragile (high‐𝑚) systems show much steeper temperature sensitivity near *T*_g_.

Although viscosity is a kinetic property, fragility is rooted in thermodynamics, being closely tied to configurational entropy and, by extension, atomic structure.^[^ ^81^ ^]^ To bridge these viewpoints, we used the microscopic volume *V*_c_, derived from viscosity modeling, as a kinetic proxy for the size of cooperatively rearranging regions in the melt. Because *V*_c_ reflects the minimal dilational volume needed for local rearrangement (not the full extent of medium‐range order) it yields a slightly smaller characteristic length than diffraction‐based metrics. For example, the spherical diameter associated with *V*_c_ at *T*_g_ is ≈0.28–0.32 nm, whereas neutron‐scattering estimates based on the first sharp diffraction peak in silicate glasses give ≈0.34–0.40 nm.^[^ ^20^ ^]^ Thus, *V*_c_ slightly underestimates medium‐range structural length scales, as expected from its kinetic definition.

**Figure 4:**
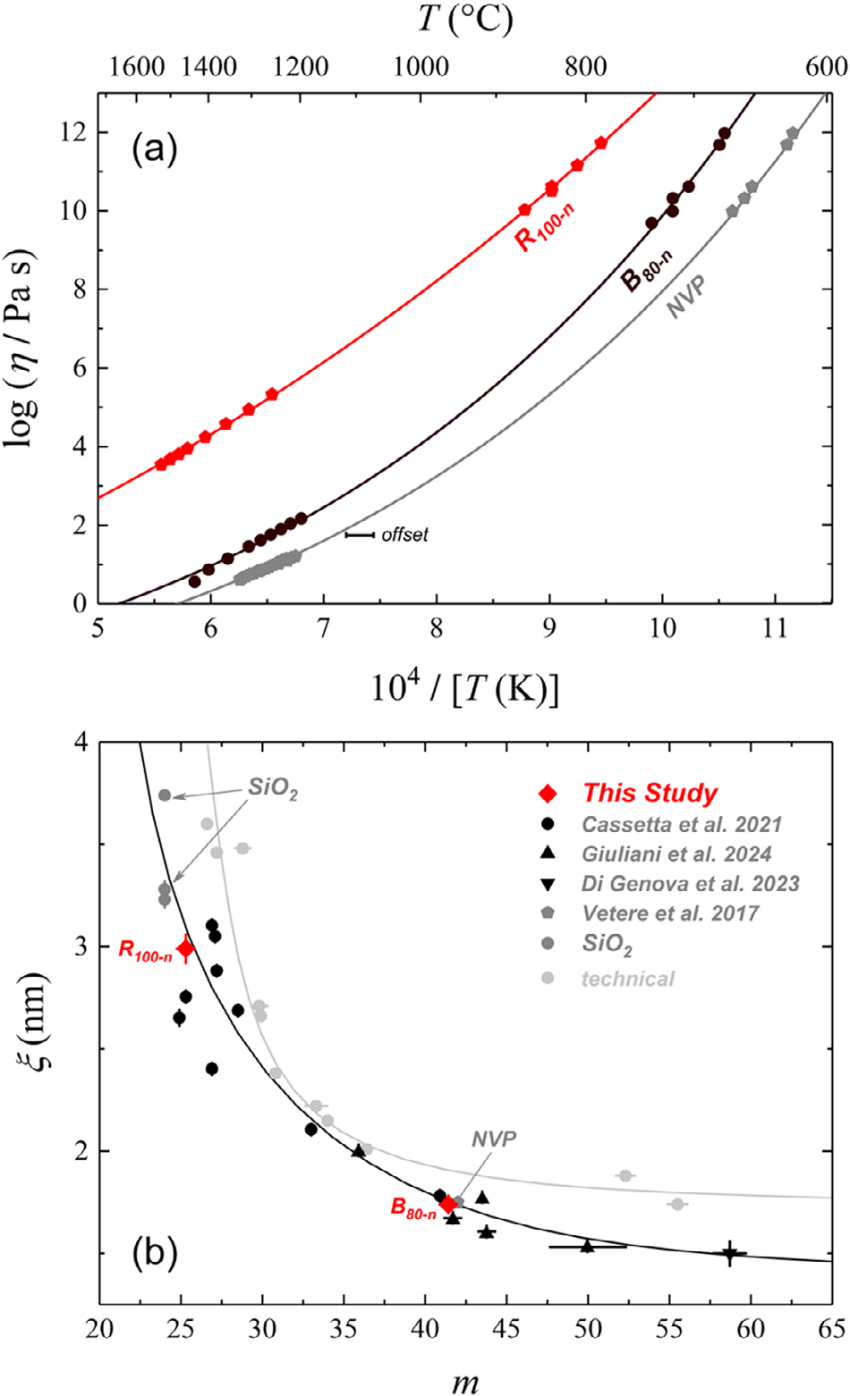
a) Temperature‐dependent viscosity of B_80‐n_, R_100‐n,_ and NVP melts (NVP curve is shifted by +0.5 unit in the 10^4^/*T* axes). Experimental values are reported in Table SI‐2 (Supporting Information). b) Relationship between the fragility index (𝑚), derived from MYEGA model fitting, and the structural correlation length (*ξ*). The plot highlights a non‐linear trend, linking melt fragility to nanoscale structural coherence.

To explore the correlation between *ξ* and 𝑚, we measured high‐ and low‐temperature viscosities of B_80‐n_, R_100‐n_, and NVP samples (data reported in Table SI‐2, Supporting Information). For NVP sample, high‐temperature viscosity data were taken from Vetere et al. 2017,^[^ ^41^ ^]^ while low‐temperature viscosities were obtained via DSC (see Methods in Section 2.5; Results in Table SI‐2, Supporting Information). Melt fragility was derived by fitting the viscosity data to the MYEGA model (Mauro‐Yue‐Ellison‐Gupta‐Allan),^[^ ^81^ ^]^ as shown in **Figure** **4**a. These values were then compared with those of the extended sample set from the literature in Figure 4b.

Figure 4b reveals a non‐linear relationship between *ξ* and 𝑚, displaying an asymptotic lower limit for *ξ* near ≈1.5 nm, corresponding to the highest fragility observed in this study (𝑚 ≈ 70), consistent with highly fragile silicate melts.^[^ ^20^ ^]^ Conversely, the upper boundary for 𝑚 aligns with the ideal strong liquid limit (𝑚 ≈ 14.9^[^ ^82^ ^]^).

**Figure 5:**
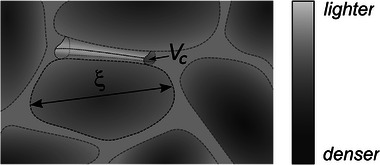
Conceptual view of nanoscale heterogeneity in volcanic glasses (granular‐medium model^[^ ^14^ ^]^). Rigid domains of characteristic size *ξ* represent structurally coherent regions with higher elastic stiffness, whereas the microscopic activation volume *V*_c_ marks compliant pockets that act as a deformable matrix between those domains. The balance between *ξ*‐scale rigid regions and *V*_c_‐sized compliant pockets govern how the melt accommodates deformation, controlling where bubble embryos form and how cavities connect to produce brittle failure.

We interpret these trends within a heterogeneous elasticity picture in which nanoscale fluctuations in rigidity govern how glass‐forming melts deform. **Figure** **5** presents a conceptual model where the glass is viewed as a granular‐like medium where rigid domains (characterized by high *ξ*) are embedded in a softer, deformable matrix (represented by *V*_c_). These heterogeneities arise from variations in bond energy density and local packing efficiency, which co‐vary as *C*_g_​, NBO/T, *ω*_BP_, and elastic moduli (notably *E*). This view is consistent with the link between viscosity and the Debye‐Waller factor 〈*u* ^2^〉, the mean‐squared atomic displacement serving as a proxy for the amplitude of oscillatory motions within the local cage formed by neighboring atoms.^[^ ^83^, ^84^ ^]^ In many glass‐formers, the viscosity is controlled by the atomic displacements through the expression *η* ∝ exp(*a*/〈*u* ^2^〉), where *a* represents the energy barrier (displacement) that must be overcome to activate viscous flow.^[^ ^85^ ^]^ Smaller 〈*u* ^2^〉 values correspond to stiffer cages, implying that the viscous flow has to overcome higher energetic barriers, thus leading to an exponential increase in viscosity.^[^ ^83^, ^86^ ^]^ The Debye–Waller factor is linked to *ω*_BP_ through *g*(*ω*)*/ω* ^2^, meaning that viscosity also scales with $\omega_{BP}^{2}$. According to Equation 11, this is proportional to the product *G*_∞_ *V*_c_, reinforcing the idea that elastic behavior and structural compactness jointly control melt dynamics. Within this framework, rhyolitic (more polymerized) melts (showing larger *ξ* and larger *V*_c_), have more uniform but stiffer cages and slower structural relaxation, which favors high viscosity and earlier brittle activation under rapid unloading. Basaltic (more depolymerized) melts (showing smaller *ξ* and smaller *V*_c_), exhibit greater local compliance and faster relaxation, promoting efficient bubble growth and deformation before brittle criteria are met.

We propose a model in which rigid, densely packed domains (high *ξ*) coexist with softer, loosely packed regions (high *V*_c_), as illustrated in Figure 5. In other words, *V*_c_ is represented as the more deformable regions (soft matrix) that separate the stiffer domains (*ξ*). In melts with high NBO/T, abundant network modifiers create numerous flexible domains, allowing local structural reorganization under stress and reducing fracture propagation. Conversely, silica‐rich rhyolites, with low NBO/T and limited heterogeneity, contain stiff, stress‐retaining regions that are prone to catastrophic brittle failure via Si–O bond rupture.

In this model, *V*_c_ is the microscopic activation volume (a kinetic measure of the local dilatation required for a rearrangement), which we use as a proxy for compliant pockets where strain concentrates. Larger *V*_c_ indicates more locally compliant regions and greater molecular mobility, favoring ductile accommodation under slow loading and lowering the barrier for bubble embryo formation. Conversely, smaller *V*_c_ marks tighter cages and reduced mobility. In volatile‐bearing magmatic liquids, *ξ* sets an effective flaw size for stress concentration (governing how cavities connect and cracks propagate), while *V*_c_ controls the local ease of cavity formation. Under rapid decompression (when deformation outpaces relaxation) the combination of *ξ*‐scale flaws and *V*_c_​‐marked compliant pockets promotes embryo nucleation and their linkage into fractures, whereas under slow loading larger *V*_c_ simply enhances viscous/ductile relaxation.

Rhyolitic melts, with low NBO/T and moderate *V*_c_ values (16.4–18.1 Å^3^), exhibit large *ξ* and high elastic energy storage potential. In combination with their high viscosity (long relaxation times), these features are consistent with a greater propensity for brittle failure and abrupt energy release under rapid decompression.^[^ ^87^, ^88^ ^]^ In contrast, basaltic melts are more plastic and capable of dissipating stress via structural reorganization. Importantly, these nano‐heterogeneities may act as structural precursor for local densification and facilitating nucleation,^[^ ^19^, ^89^ ^]^ an effect particularly discussed for Fe‐Ti rich melts.^[^ ^4^, ^90^ ^]^

Samples R_100‐n_ and R_100_, despite having the same or nearly identical chemical composition, exhibit distinct physical behaviors. R_100‐n_ shows a higher *T*_g_ and slightly higher elastic moduli, indicating a more cohesive network, further supported by its higher density. This trend extends to the vibrational density of states (VDoS), where R_100‐n_ displays a BP at slightly higher energies compared to R_100_. Correspondingly, *ξ* and *V_c_* ​ are slightly lower in R_100‐n_.

These differences appear to be driven by the oxidation state of iron. Raman spectra (Figure SI‐2, Supporting Information) show a significantly more intense Fe^3+^ band in R_100‐n_, implying a higher Fe^3+^/Fe^2+^ ratio. This, regardless of the specific coordination state, typically stiffens the network via stronger Fe–O bonding and more effective charge balancing, contributing to the higher *T*_g_, enhanced elastic properties, and upshift of VDoS. This interpretation is consistent with lower *C*_g_ value in R_100_, indicative of a more open, less densely packed network where Fe‐related interactions are comparatively weaker.^[^ ^91^ ^]^

While the dynamics of volcanic eruptions arise from a complex interplay of gas content, crystallinity, decompression rate, and conduit processes, this study offers a structural perspective that complements existing models. In line with prior work emphasizing the central role of melt properties in eruptive behavior,^[^ ^1^ ^]^ we present new evidence that the nanoscale descriptors of silicate melts, particularly the relationship between *V*_c_ ​and *ξ*, help rationalize how silicate melts accommodate stress, and where they are prone to initiate cavities, and how those cavities connect. In particular, these parameters can bias the efficiency of bubble nucleation and fragmentation, even though melt structure alone does not determine eruptive style.

Finally, since bubble nucleation involves nanoscale dynamics,^[^ ^25^, ^92^ ^]^ it is reasonable that both the degree and spatial scale of elastic heterogeneity (captured by *V*_c_ and *ξ*) influence the availability of favorable sites for embryo formation and coalescence,^[^ ^24^, ^93^ ^]^ which provides a transferable bridge between glass vibrational structure and magmatic rheology.

Building on this structural foundation, we now outline how our nanoscale descriptors relate, in a first‐order sense, to degassing and failure. The correlation length *ξ* and the microscopic activation volume *V*_c_​ indicate, respectively, the scale of stress concentrators and the presence of locally compliant regions; together, they help frame where cavities may form and how they may connect under volcanic loading. We present this link conservatively, as guidance for integrating melt structure into broader eruptive models.

#### 3.4.1 Linking *ξ* and *V*_c_ to Magma Degassing and Fragmentation

At the onset of degassing, bubble embryos are expected to form preferentially within *V*_c_‐sized compliant regions, where a lower local shear stiffness reduces both the critical radius (r*) and the free‐energy barrier (ΔG*) from classical nucleation theory (CNT). Along *ξ*‐scale elastic‐contrast interfaces, cavities coalesce and cracks initiate more readily because these interfaces act as effective flaws. Whether this pathway culminates in brittle fragmentation depends on the competition between the process timescale and the material relaxation time *τ*  =  *η*/*G* (Maxwell relation; *η* viscosity, *G* shear modulus).^[^ ^94^ ^]^ In rhyolites, high *η* lengthens *τ*, so decompression often outpaces relaxation; the medium responds elastically, overpressure is sustained, and brittle failure occurs at lower decompression rates. In basalts, low *η* shortens *τ*, promoting bubble growth and open‐system degassing that dissipate overpressure before failure.

For bubble nucleation, CNT gives the standard forms,

(12) $$r^{*}=\frac{2\gamma }{\Delta P}\;,\Delta G^{*}=\frac{16\pi \gamma^{3}}{3\Delta P^{2}}\;,$$

where *γ* is the melt–gas surface tension and Δ*P* the supersaturation (driving pressure).^[^ ^95^, ^96^, ^97^ ^]^ When nucleation occurs faster than the melt can relax (an elastic response), several treatments capture the elastic penalty via an effective driving

(13) $$\Delta P_{\mathrm{eff}}\approx \Delta P-\beta \;G_{\mathrm{loc}}$$

with *G*_loc_ the local shear modulus and *β*  ≈  1 a geometry factor; softer pockets (lower *G*_loc_) yield larger Δ*P*_eff_ and thus smaller r*and ΔG*.^[^ ^5^, ^98^ ^]^

For fragmentation, elastic‐contrast interfaces behave as flaws of size ∼ *ξ*. Linear‐elastic fracture mechanics gives the failure stress scaling as:

(14) $$\sigma_{c}\approx \sqrt{\frac{E\;\Gamma }{\pi \;\xi }}$$

where *E* is Young's modulus and Γ the fracture surface energy.^[^ ^99^ ^]^ Larger *ξ* (and lower *E*) lowers σ_*c*_; once deformation outpaces relaxation, *ξ*‐scale flaws become active and foster cavity linkage and crack propagation.

In this first‐order picture, *V*_c_ biases where embryos start, while *ξ* sets how cavities connect and fail. Across our series, both *ξ* and *V*_c_ increase from basalt to rhyolite, a combination consistent with enhanced local nucleation and reduced brittle strength in silicic melts under rapid unloading, in qualitative agreement with rapid‐decompression observations.^[^ ^100^, ^101^ ^]^ We emphasize that these links are complementary to other controls (H_2_O content and speciation, crystallinity, ascent rate, conduit processes). For example, dissolved water can soften the network (i.e., OH depolymerization) and thus modify both relaxation and thresholds.^[^ ^102^ ^]^ Accordingly, we view *ξ* and *V*_c_ as structural descriptors that help set thresholds and identify to which eruptive behavior the magma is more prone, not as deterministic predictors of eruptive style.

#### 3.4.2 Context from Technical Glasses: Similarities and Departures

When technical glasses are added, several trends diverge from the volcanic patterns. In the *ξ* vs. NBO/T plot (Figure 3a), binary Na_2_O–SiO_2_ (NS) and K_2_O–SiO_2_ (KS), together with mineral analogs, indicate a strong silica‐network control on *ξ*. Soda–lime standards (SRM‐710, DGG‐1), which lack Al and Fe, cluster at comparatively higher *ξ* for a given NBO/T. This likely reflects the absence of network intermediates (Al, Fe) that, in volcanic compositions, contribute to connectivity and elastic heterogeneity, though a targeted study of their coordination/redox would be needed to test this hypothesis. More broadly, the presence (or absence) of Al_2_O_3_ and Fe_2_O_3_ (tetrahedral Fe^3+^) is consistent with shifts toward larger *ξ* (≈2–4 nm vs. <2 nm in modifier‐rich binaries), owing to a higher fraction of tetrahedral units in the medium‐range structure rather than interstitial ionic species. In soda–lime formulations, Na_2_O is deliberately elevated (≉10–20 wt.%) to lower working temperature and suppress crystallization, while Al_2_O_3_ is kept <1–2 wt.% to avoid raising viscosity, choices that naturally bias *ξ* and NBO/T relative to volcanic chemistries.

Extending the comparison to *ξ* vs. *m* (Figure 5b), the overall shape is similar, but technical glasses diverge at the extremes. For a given m, Fe‐ and Al‐free systems tend to show higher *ξ*, plausibly because their networks are dominated by Si–O–Si ring topologies.^[^ ^20^ ^]^ These rigid bridges confer local stiffness yet can enhance nanoscale fluctuations, as the elastic response depends on ring size and connectivity.^[^ ^50^, ^103^, ^104^ ^]^ Where formers/intermediates and modifiers coexist at intermediate levels, the datasets overlap more, suggesting that medium‐range order, rather than a single short‐range motif, governs the apparent *ξ* vs. *m* relation.

**Figure 6:**
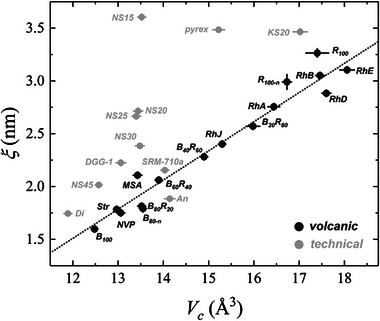
Linear dependence between the correlation length (*ξ*) and the microscopic volume (*V_c_*) for volcanic glasses ranging from basalt (B_100_) to rhyolite (R_100_) in dark gray and for technical glasses set (light gray). Data of Str, NVP, MSA, Rh‐series, and technical are reported in the SI.

The *ξ* *vs.* *V*_c_ plot (**Figure** **6**) further clarifies these differences. Volcanic glasses cluster tightly along a common trend, whereas technical compositions deviate systematically. Diopside (Di) and Anorthite (An), rich in divalent cations, plot close to the volcanic line, consistent with faster acoustic propagation and a stiffer elastic response. Soda‐lime standards (DGG‐1, SRM‐710) also fall reasonably close, likely reflecting the lack of Al/Fe contributions to connectivity and elastic heterogeneity. The binary NS and KS (soda‐potassa silicate series) show similar offsets, consistent with simplified chemistries and fewer network formers. Pyrex (borosilicate) departs for a different reason: boron introduces distinct structural units and bidimensional motifs that create elastic “soft spots,” weakening the correlation seen in silicate‐only systems. Overall, these departures indicate that while the volcanic dataset is internally consistent, technical glasses (with narrower chemistries or additional network formers) sample different balances of elastic heterogeneity and cooperative volume, explaining their deviations and reinforcing the distinct behavior of volcanic glass‐forming melts.

## 4 Conclusion

This study presents a structural perspective on volcanic glasses, exploring how nanoscale heterogeneities relate to the physical properties of the melts. By examining a compositional series of silicate glasses from basaltic to rhyolitic end‐members, we assess the variability of three key structural parameters: correlation length (*ξ*), microscopic free volume (*V*_c_), and melt fragility (𝑚) and consider how these may be linked to differences in rheological behavior.

Our results indicate that nanoscale elastic inhomogeneity helps set where degassing starts and when brittle failure can occur. The boson‐peak–derived *ξ* (size of comparatively stiffer melt domains) and *V*_c_ (size of compliant pockets where local dilatation concentrates) act together to bias nucleation and failure thresholds in the melt, the continuous phase that accommodates deformation and ultimately breaks. Bubble embryos form preferentially in *V*_c_‐sized compliant regions and along *ξ*‐scale interfaces where stress focuses; melts with larger *V*_c_ and *ξ* therefore show enhanced local nucleation. The same *ξ*‐scale interfaces behave as effective flaws that promote cavity coalescence and crack growth when deformation outpaces relaxation, consistent with lower fragmentation thresholds in more polymerized (rhyolitic) compositions. For crystals, *V*_c_ facilitates the cooperative rearrangements needed to initiate ordering upon undercooling, while *ξ* provides elastic contrasts that may pin or template early clusters; in both cases, nanoscale heterogeneity shifts rates and onset conditions rather than phase equilibria.

Crystals and volatiles remain essential co‐controls through their effects on effective viscosity, elasticity, and permeability. Within that broader context, *ξ* and *V*_c_ provide melt‐scale descriptors that help integrate glass vibrational structure into models of degassing and fragmentation, clarifying how nanoscale structure contributes to eruptive dynamics.

## Conflict of Interest

The authors declare no conflict of interest.

## Supporting information



Supporting Information

## Data Availability

The data that support the findings of this study are available from the corresponding author upon reasonable request.
