# Supplementary material for: Chemically Driven Nano‐Elastic Heterogeneities Control Fragility in Volcanic Melts
Source: Adv Sci (Weinh). 2025 Nov 8;13(5):e12063. doi: 10.1002/advs.202512063 (PMC12850459; doi:10.1002/advs.202512063)
Supplement: Supplementary file 1 — Supporting Information [file ADVS-13-e12063-s001.docx]

**Chemically Driven Nano-elastic Heterogeneities Control Fragility in Volcanic Glass-Forming Melts**

***SUPPLEMENTARY INFORMATION***

Michele Cassetta*^,1,2^, Daria Szewczyk^3^, Gabriele Giuliani^4^, Serena Dominijanni^5^, Francesco Vetere^6^, Gianluca Iezzi^7,8,9^, Francesco Radica^7,8^, Dmitry Bondar^5,10^, Nicola Daldosso^1^, Fabrizio Di Fiore^11^, Alessio Pontesilli^11^, Hideyuki Mizuno^12^, Danilo Di Genova^5^

*1-﻿ Department of Engineering for Innovation Medicine, University of Verona, I-37134 Verona, Italy*

*2- ﻿Department of Earth Science, University of Torino, I-10125 Torino, Italy*

*3- Division of Low Temperature and Superconductivity, Institute of Low Temperature and Structure Research PAS, Okólna 2, P-50422 Wrocław, Poland*

*4-* *Dipartimento di Scienze, Università degli Studi Roma Tre, I-00146 Roma, Italy*

*5- Institute of Science, Technology and Sustainability for Ceramics (ISSMC) of the National Research Council (CNR), Via Granarolo 64, I-48018, Faenza, Italy*

*6- ﻿Department of Physical Sciences, Earth and Environment, University of Siena, I-53100 Siena, Italy*

*7- Dipartimento di Ingegneria e Geologia, Universita` degli studi di Chieti, I-66100 Chieti, Italy
8- UdA-TechLab, Research Center, University “G. d’Annunzio” of Chieti-Pescara, 66100 Chieti, Italy
9- CNR - Istituto Officina dei Materiali (IOM) c/o Department of Physics and Geology, University of Perugia, I-06123 Perugia, Italy*

*10 - Bayerisches Geoinstitut, University of Bayreuth, Universitätsstraße 30, D-95447 Bayreuth, Germany*

*11- ﻿Istituto Nazionale di Geofisica e Vulcanologia, Sezione di Roma 1, I-00143 Roma, Italy*

*12 - Graduate School of Arts and Sciences, The University of Tokyo, 153-8902 Tokyo, Japan*

*corresponding author: Michele Cassetta [michele.cassetta@univr.it](mailto:michele.cassetta@univr.it) or [michele.cassetta@unito.it](mailto:michele.cassetta@unito.it)

| ***sample*** | **SiO_2_** | **TiO_2_** | **Al_2_O_3_** | **FeO_(t)_** | **MnO** | **MgO** | **CaO** | **Na_2_O** | **K_2_O** | **P_2_O_5_** | **B_2_O_3_** | **NBO/T** | ***Ref.*** |
| --- | --- | --- | --- | --- | --- | --- | --- | --- | --- | --- | --- | --- | --- |
| ***volcanic*** | | | | | | | | | | | | | |
| ***B_100_*** | 48.02 | 0.98 | 15.59 | 10.23 | 0.18 | 9.42 | 13.20 | 1.79 | 0.04 | 0.06 | - | 0.88 | ^[1]^ |
| ***B_80_R_20_*** | 53.01 | 0.80 | 14.99 | 8.54 | 0.15 | 7.58 | 10.79 | 2.18 | 1.02 | 0.02 | - | 0.68 | ^[1]^ |
| ***B_60_R_40_*** | 57.97 | 0.65 | 14.62 | 6.95 | 0.13 | 5.81 | 8.46 | 2.59 | 1.99 | 0.04 | - | 0.50 | ^[1]^ |
| ***B_40_R_60_*** | 62.73 | 0.46 | 14.05 | 5.42 | 0.12 | 4.01 | 6.07 | 2.95 | 3.02 | 0.02 | - | 0.34 | ^[1]^ |
| ***B_20_R_80_*** | 67.91 | 0.29 | 13.59 | 3.69 | 0.11 | 2.18 | 3.63 | 3.29 | 3.99 | 0.02 | - | 0.19 | ^[1]^ |
| ***R_100_*** | 73.97 | 0.12 | 13.48 | 2.06 | 0.08 | 0.44 | 1.36 | 3.75 | 4.89 | 0.03 | - | 0.06 | ^[1]^ |
| ***B_80-n_*** | 52.98 | 0.81 | 15.13 | 8.29 | 0.18 | 6.92 | 10.89 | 2.11 | 0.95 | 0.03 | - | 0.69 | *^t.w.^* |
| ***R_100-n_*** | 72.34 | 0.13 | 13.48 | 2.18 | 0.11 | 0.45 | 1.36 | 3.60 | 4.65 | 0.03 | - | 0.06 | *^t.w.^* |
| ***SiO_2_*** | 100 | - | - | - | - | - | - | - | - | - | - | 0.00 | ^[2]^ |
| ***RhA*** | 77.63 | 0.11 | 12.73 | 3.03 | 0.03 | 0.06 | 0.92 | 4.44 | 1.62 | - | - | 0.03 | ^[2]^ |
| ***RhB*** | 77.28 | 0.14 | 13.39 | 2.94 | 0.02 | 0.06 | 0.75 | 2.71 | 3.61 | 0.03 | - | 0.01 | ^[2]^ |
| ***RhD*** | 76.83 | 0.11 | 12.43 | 2.96 | 0.05 | 0.07 | 0.9 | 2.93 | 4.29 | 0.04 | - | 0.04 | ^[2]^ |
| ***RhE*** | 75.33 | 0.12 | 13.61 | 2.93 | 0.03 | 0.07 | 0.88 | 1.41 | 6.80 | 0.06 | - | 0.03 | ^[2]^ |
| ***RhH*** | 77.25 | 0.10 | 11.95 | 2.62 | 0.06 | 0.22 | 1.08 | 3.23 | 4.35 | 0.05 | - | 0.06 | ^[2]^ |
| ***RhI*** | 76.24 | 0.06 | 11.46 | 2.85 | 0.03 | 0.44 | 1.31 | 2.99 | 3.83 | 0.04 | - | 0.07 | ^[2]^ |
| ***RhJ*** | 73.75 | 0.31 | 11.99 | 3.31 | 0.07 | 1.64 | 2.98 | 3.16 | 3.56 | 0.02 | - | 0.15 | ^[2]^ |
| ***MSA*** | 59.58 | 0.58 | 17.94 | 6.28 | 0.20 | 2.86 | 7.71 | 3.75 | 0.84 | - | - | 0.28 | ^[2]^ |
| ***Str*** | 49.30 | 0.86 | 16.90 | 8.09 | 0.16 | 6.12 | 12.00 | 2.74 | 2.14 | 0.5 | - | 0.66 | ^[2]^ |
| ***S34F0*** | 50.86 | 0.03 | 6.63 | - | - | 35.30 | 7.14 | 0.02 | 0.02 | - | - | 1.92 | ^[3]^ |
| ***S45F7*** | 48.50 | 0.01 | 6.47 | 8.22 | - | 29.85 | 6.87 | 0.03 | 0.03 | - | - | 1.96 | ^[3]^ |
| ***S44F6*** | 48.16 | 0.01 | 6.48 | 8.52 | - | 30.03 | 6.74 | 0.02 | 0.03 | - | - | 1.98 | ^[3]^ |
| ***S44F12+*** | 46.50 | 0.01 | 6.15 | 15.61 | - | 25.08 | 6.59 | - | 0.02 | - | - | 2.00 | ^[3]^ |
| ***S44F12*** | 46.53 | 0.02 | 6.01 | 15.83 | - | 24.97 | 6.58 | 0.02 | 0.02 | - | - | 2.01 | ^[3]^ |
| ***S41F6*** | 49.27 | 0.01 | 4.05 | 8.84 | - | 36.95 | 3.52 | 0.02 | 0.01 | - | - | 2.57 | ^[3]^ |
| ***S45F7*** | 49.27 | 0.01 | 3.96 | 8.65 | - | 34.76 | 3.31 | 0.02 | 0.02 | - | - | 2.24 | ^[3]^ |
| ***S34F0*** | 44.91 | 0.03 | 7.78 | 0.17 | - | 29.37 | 17.63 | 0.02 | 0.07 | - | - | 2.04 | ^[3]^ |
| ***C10M4*** | 50.76 | 0.84 | 17.47 | 6.03 | 0.14 | 4 | 10.08 | 2.95 | 7.18 | 0.55 | - | 0.42 | ^[4]^ |
| ***C18M5*** | 46.12 | 0.74 | 15.62 | 5.5 | 0.13 | 4.5 | 18.09 | 2.6 | 6.24 | 0.45 | - | 0.74 | ^[4]^ |
| ***C26M5*** | 41.49 | 0.68 | 13.66 | 4.81 | 0.12 | 4.91 | 26.35 | 2.2 | 5.33 | 0.44 | - | 1.14 | ^[4]^ |
| ***C13M8*** | 46.67 | 0.77 | 15.9 | 5.46 | 0.12 | 8.29 | 13.1 | 2.68 | 6.5 | 0.5 | - | 0.74 | ^[4]^ |
| ***C19M17*** | 38.8 | 0.65 | 12.95 | 4.92 | 0.12 | 17.02 | 19.15 | 1.82 | 4.15 | 0.42 | - | 1.55 | ^[4]^ |
| ***NVP*** | 61.48 | 0.36 | 8.95 | - | - | 14.12 | 6.81 | 8.85 | 0.21 | - | - | 0.88 | ^[5]^ |
| ***technical*** | | | | | | | | | | | | | |
| ***Di*** | 55.35 | - | - | - | - | 18.46 | 26.19 | - | - | - | - | 2.01 | ^[2]^ |
| ***An*** | 43.19 | - | 36.65 | - | - | - | 20.16 | - | - | - | - | - | ^[2]^ |
| ***DGG-1*** | 71.72 | 0.14 | 1.23 | - | - | 4.18 | 6.73 | 14.95 | 0.38 | - | - | 0.75 | ^[2]^ |
| ***SRM-710*** | 70.50 | - | - | - | - | - | 11.60 | 8.70 | 7.70 | - | - | 0.68 | ^[6]^ |
| ***pyrex*** | 81.10 | 0.02 | 2.05 | - | - | 0.03 | 0.05 | - | - | - | 12.30 | 0.10 | ^[7]^ |
| ***NS15*** | 85.44 | - | - | - | - | - | - | 14.66 | - | - | - | 0.33 | ^[8]^ |
| ***NS20*** | 79.56 | - | - | - | - | - | - | 20.95 | - | - | - | 0.51 | ^[8]^ |
| ***NS25*** | 75.07 | - | - | - | - | - | - | 24.35 | - | - | - | 0.63 | ^[8]^ |
| ***NS30*** | 63.56 | - | - | - | - | - | - | 36.44 | - | - | - | 1.11 | ^[8]^ |
| ***NS45*** | 53.96 | - | - | - | - | - | - | 46.04 | - | - | - | 1.65 | ^[8]^ |
| ***KS20*** | 71.84 | - | - | - | - | - | - | - | 28.16 | - | - | 0.50 | ^[9]^ |

**Table SI-1.** Chemical composition in wt.% and NBO/T values of the studied glasses (t.w. stands for this work)

| ***R_100-n_*** | | ***B_80-n_*** | | ***NVP*** | |  |
| --- | --- | --- | --- | --- | --- | --- |
| ***T*** | **log** $\boldsymbol{\eta}$ | ***T*** | **log** $\boldsymbol{\eta}$ | ***T*** | **log** $\boldsymbol{\eta}$ |  |
| (°C) | (Pa s) | (°C) | (Pa s) | (°C) | (Pa s) |  |
| 1525 | 3.54 | 1434 | 0.56 | 665.3 | 11.99 | ***DSC*** |
| 1500 | 3.68 | 1399 | 0.87 | 698.2 | 10.62 |  |
| 1476 | 3.81 | 1353 | 1.15 | 715.0 | 9.99 |  |
| 1453 | 3.95 | 1305 | 1.46 | 669.9 | 11.67 |  |
| 1407 | 4.24 | 1279 | 1.62 | 704.7 | 10.32 |  |
| 1357 | 4.58 | 1258 | 1.76 | 1326 | 1.21 | ***CC*** ^[10]^ |
| 1305 | 4.94 | 1237 | 1.90 | 1336 | 1.17 |  |
| 1255 | 5.33 | 1218 | 2.04 | 1344 | 1.10 |  |
| 865.7 | 10.02 | 1197 | 2.17 | 1347 | 1.15 |  |
| 835.4 | 10.51 | 674.5 | 11.98 | 1356 | 1.11 |  |
| 835.3 | 10.62 | 704.0 | 10.62 | 1365 | 1.01 |  |
| 808.3 | 11.16 | 717.80 | 9.99 | 1378 | 0.99 |  |
| 784.2 | 11.73 | 678.7 | 11.68 | 1388 | 0.94 |  |
|  |  | 717.7 | 10.32 | 1397 | 0.90 |  |
|  |  | 736.40 | 9.69 | 1407 | 0.86 |  |
|  |  |  |  | 1413 | 0.83 |  |
|  |  |  |  | 1417 | 0.83 |  |
|  |  |  |  | 1427 | 0.78 |  |
|  |  |  |  | 1437 | 0.74 |  |
|  |  |  |  | 1437 | 0.76 |  |
|  |  |  |  | 1446 | 0.70 |  |
|  |  |  |  | 1456 | 0.66 |  |
|  |  |  |  | 1462 | 0.63 |  |
|  |  |  |  | 1462 | 0.60 |  |

**Table SI-2.** Viscosity data for B_80-n_ and R_100-n_ obtained by concentric cylinder (CC) and micro-penetration (MP) techniques and for NVP low-*T* viscosity data were obtained by shift factor of Ref.^[10]^ using DSC while high-*T* viscosity data are from Ref.^[5]^.

| ***Sample*** | ***Wt*** | ***L_1_*** | ***L_2_*** | ***Tck*** | ***V*** | ***ρ*** |
| --- | --- | --- | --- | --- | --- | --- |
|  | (g) | (mm) | (mm) | (mm) | (cm^3^) | (g cm^-3^) |
| ***B_100_*** | 0.062 | 4.790 | 4.800 | 0.960 | 0.022 | 2.804 |
| ***B_80_R_20_*** | 0.058 | 4.890 | 3.370 | 1.320 | 0.022 | 2.648 |
| ***B_60_R_40_*** | 0.063 | 4.500 | 4.080 | 1.310 | 0.024 | 2.599 |
| ***B_40_R_60_*** | 0.055 | 5.050 | 3.360 | 1.320 | 0.022 | 2.460 |
| ***B_20_R_80_*** | 0.077 | 5.270 | 4.660 | 1.320 | 0.032 | 2.366 |
| ***R_100_*** | 0.084 | 6.230 | 6.240 | 0.970 | 0.038 | 2.238 |

**Table SI-3** Density determination of the isotropic glass prisms.

| ***Sample*** | ***ω_BP,Raman_*** | ***ω_BP,VDoS_*** | ***v_t_*** | ***ξ*** | ***V_c_*** | ***m*** | ***Ref.*** |
| --- | --- | --- | --- | --- | --- | --- | --- |
|  | [cm^-1^] | [cm^-1^] | [m s^-1^] | [nm] | [Å^3^] | - |  |
| ***volcanic*** | | | | | | | |
| ***B_100_*** | 84.9 (8) | 75.8 (7) | 3629 (12) | 1.60 (2) | 12.48 (7) | - | *^t.w.^* |
| ***B_80_R_20_*** | 75.3 (4) | 66.1 (4) | 3592 (8) | 1.81 (1) | 13.52 (9) | - | *^t.w.^* |
| ***B_60_R_40_*** | 67.4 (4) | 58.1 (3) | 3587 (16) | 2.06 (1) | 13.90 (8) | - | *^t.w.^* |
| ***B_40_R_60_*** | 61.7 (5) | 52.3 (4) | 3574 (8) | 2.28 (2) | 14.90 (8) | - | *^t.w.^* |
| ***B_20_R_80_*** | 55.6 (6) | 46.1 (5) | 3550 (10) | 2.57 (3) | 15.98 (15) | - | *^t.w.^* |
| ***R_100_*** | 45.8 (5) | 36.1 (4) | 3534 (9) | 3.26 (4) | 17.41 (25) | - | *^t.w.^* |
| ***SiO_2_*** | 48.5 (5) | 38.8 (4) | 3769 (13) | 3.23 (4) | 21.65 (22) | 24.0 (5) | ^[2]^ |
| ***B_80-n_*** | 76.3 (4) | 67.1 (3) | 3609 (9) | 1.79 (1) | 13.55 (8) | 41.7 (3) | *^t.w.^* |
| ***R_100-n_*** | 49.2 (4) | 39.6 (9) | 3547 (9) | 2.99 (7) | 16.73 (12) | 25.2 (1) | *^t.w.^* |
| ***RhA*** | 53.2 (5) | 43.6 (4) | 3604 (13) | 2.75 (3) | 16.44 (13) | 25.3 (1) | ^[2]^ |
| ***RhB*** | 49.1 (4) | 39.5 (3) | 3610 (8) | 3.05 (2) | 17.46 (10) | 27.1 (4) | ^[2]^ |
| ***RhD*** | 51.2 (2) | 41.6 (2) | 3595 (8) | 2.88 (2) | 17.60 (10) | 27.2 (3) | ^[2]^ |
| ***RhE*** | 47.8 (3) | 38.2 (2) | 3550 (14) | 3.10 (2) | 18.06 (16) | 26.9 (2) | ^[2]^ |
| ***RhH*** | 54.1 (4) | 44.6 (3) | 3590 (11) | 2.69 (2) | - | 28.5 (2) | ^[2]^ |
| ***RhI*** | 54.7 (5) | 45.2 (4) | 3590 (14) | 2.65 (3) | - | 24.9 (1) | ^[2]^ |
| ***RhJ*** | 59.2 (5) | 49.7 (4) | 3581 (7) | 2.40 (2) | 15.27 (8) | 26.9 (1) | ^[2]^ |
| ***MSA*** | 66.5 (5) | 57.1 (4) | 3608 (12) | 2.11 (2) | 13.43 (10) | 33.0 (2) | ^[2]^ |
| ***Str*** | 76.0 (5) | 66.8 (4) | 3569 (12) | 1.78 (1) | 12.97 (9) | 40.9 (2) | ^[2]^ |
| ***S44F0*** | 93.02 (6) | 84.1 (5) | 3826 (1) | 1.52 (1) | - | - | ^[11]^ |
| ***S45F7*** | 94.3 (7) | 85.3 (6) | 3757 (3) | 1.47 (1) | - | - | ^[11]^ |
| ***S44F6*** | 91.7 (6) | 82.7 (5) | 3718 (3) | 1.50 (1) | - | - | ^[11]^ |
| ***S44F12+*** | 92.5 (8) | 83.5 (7) | 3627 (3) | 1.45 (1) | - | - | ^[11]^ |
| ***S44F12*** | 87.3 (10) | 78.3 (9) | 3636 (3) | 1.55 (2) | - | - | ^[11]^ |
| ***S41F6*** | 94.1 (6) | 85.2 (5) | 3755 (7) | 1.47 (1) | - | - | ^[11]^ |
| ***S45F7*** | 89.3 (7) | 80.3 (6) | 3736 (4) | 1.55 (1) | - | - | ^[11]^ |
| ***S34F0*** | 96.6 (5) | 87.7 (5) | 3819 (9) | 1.45 (1) | - | - | ^[11]^ |
| ***C10M4*** | 67.9 (4) | 58.6 (3) | 3502 (5) | 2.00 (1) | - | 35.9 (1) | ^[4]^ |
| ***C18M5*** | 75.6 (5) | 66.4 (4) | 3512 (11) | 1.76 (1) | - | 43.5 (4) | ^[4]^ |
| ***C26M5*** | 86.1 (8) | 77.0 (7) | 3526 (15) | 1.53 (2) | - | 50.0 (2.3) | ^[4]^ |
| ***C13M8*** | 80.1 (6) | 71.0 (5) | 3540 (8) | 1.66 (1) | - | 41.7 (3) | ^[4]^ |
| ***C19M17*** | 84.7 (6) | 75.6 (5) | 3620 (9) | 1.60 (1) | - | 43.8 (5) | ^[4]^ |
| ***NVP*** | 78.3 (4) | 69.1 (4) | 3623 (8) | 1.75 (1) | 13.09 (7) | 41.5 (1) | ^[5]^ |
| ***technical*** | | | | | | | |
| ***Di*** | 90.0 (5) | 81.0 (5) | 3741 (15) | 1.74 (1) | 11.89 (9) | 55.5 (4) | ^[2]^ |
| ***An*** | 85.0 (5) | 75.9 (5) | 3753 (15) | 1.88 (1) | 14.14 (12) | 52.3 (4) | ^[2]^ |
| ***DGG-1*** | 70.6 (9) | 61.3 (9) | 3451 (13) | 2.22 (3) | 13.06 (11) | 33.3 (6) | ^[2]^ |
| ***SRM-710a*** | 70.0 (1.5) | 60.7 (1.5) | 3303 (10) | 2.15 (2) | 14.03 (9) | 34.0 (1) | ^[6,12]^ |
| ***pyrex*** | 52.1 (3) | 42.5 (3) | 3422 (15) | 3.48 (2) | 15.22 (13) | 28.8 (3) | ^[7,13]^ |
| ***NS15*** | 51.0 (1) | 41.4 (1) | 3421 (10) | 3.60 (3) | 13.52 (9) | 26.6 (1) | ^[8]^ |
| ***NS20*** | 60.1 (7) | 50.6 (7) | 3330 (10) | 2.71 (3) | 13.44 (10) | 29.8 (2) | ^[8]^ |
| ***NS25*** | 60.0 (7) | 50.5 (7) | 3257 (10) | 2.66 (2) | 13.40 (10) | 29.9 (3) | ^[8]^ |
| ***NS30*** | 63.8 (8) | 54.4 (8) | 3204 (10) | 2.38 (2) | 13.48 (10) | 30.8 (2) | ^[8]^ |
| ***NS45*** | 71.0 (1) | 61.7 (1) | 3154 (10) | 2.01 (2) | 12.57 (8) | 36.4 (2) | ^[8]^ |
| ***KS20*** | 48.0 (8) | 38.4 (8) | 2970 (15) | 3.46 (2) | 17.02 (16) | 27.2 (3) | ^[14–17]^ |

**Table SI-4** Boson peak position in function of ***I^red^*** derived by low-frequency Raman spectroscopy (***ω_BP,Raman_***) and corrected by pure vibrations (***ω_BP,VDoS_***), transverse sound velocity (***v_t_***) derived by Brillouin spectroscopy, correlation length (***ξ***) obtained by Eq.9 and microscopic volume (***V_c_***) obtained by Eq. 11. (t.w. stands for this work).


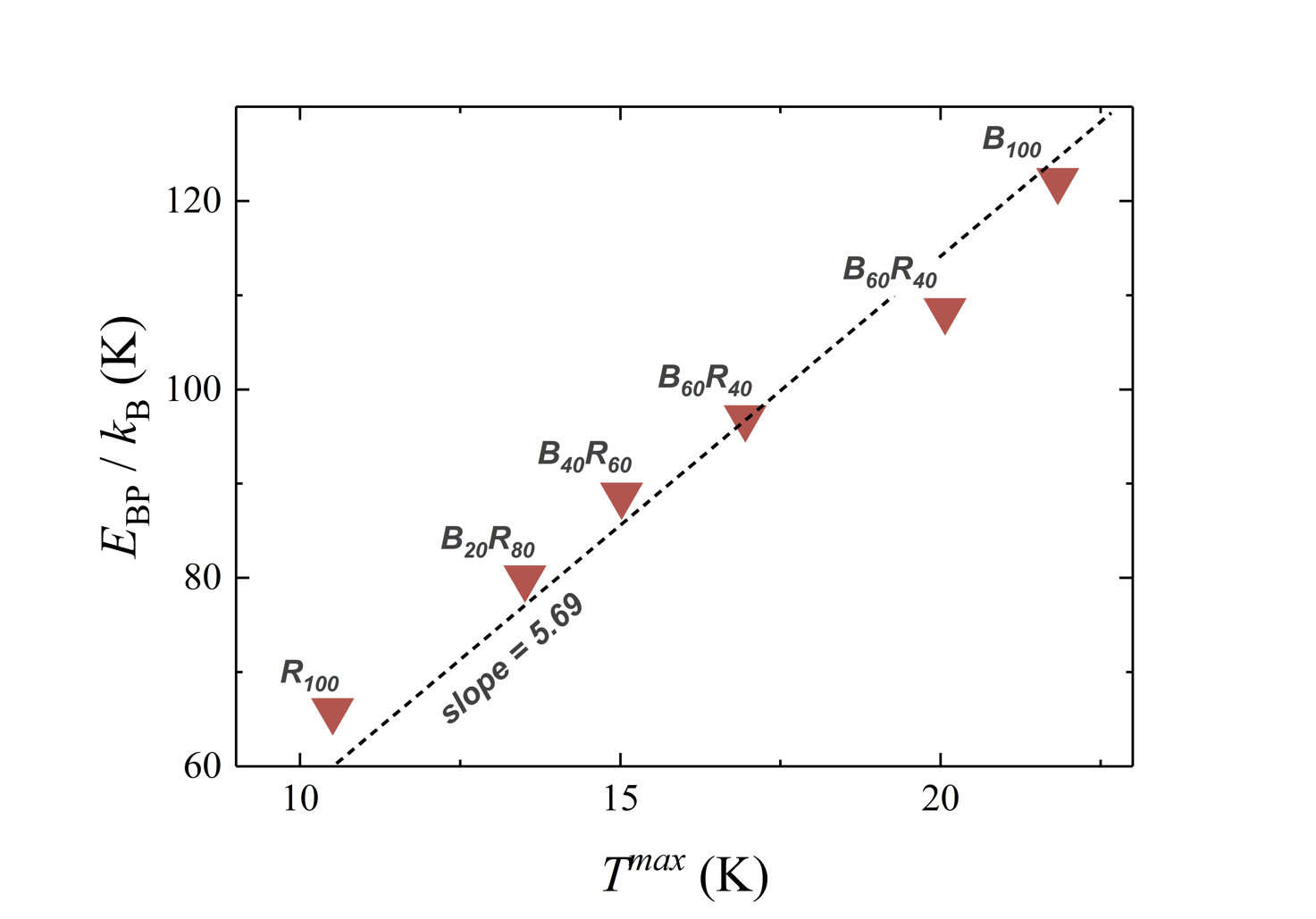


**Figure SI-1** Plot of the Boson peak energy *E_BP_* *vs.* *T^max^* (temperature of *C*_p_-*C*_TLS_*T*/*T*^3^ maximum) for B_100_-R_100_ glasses.


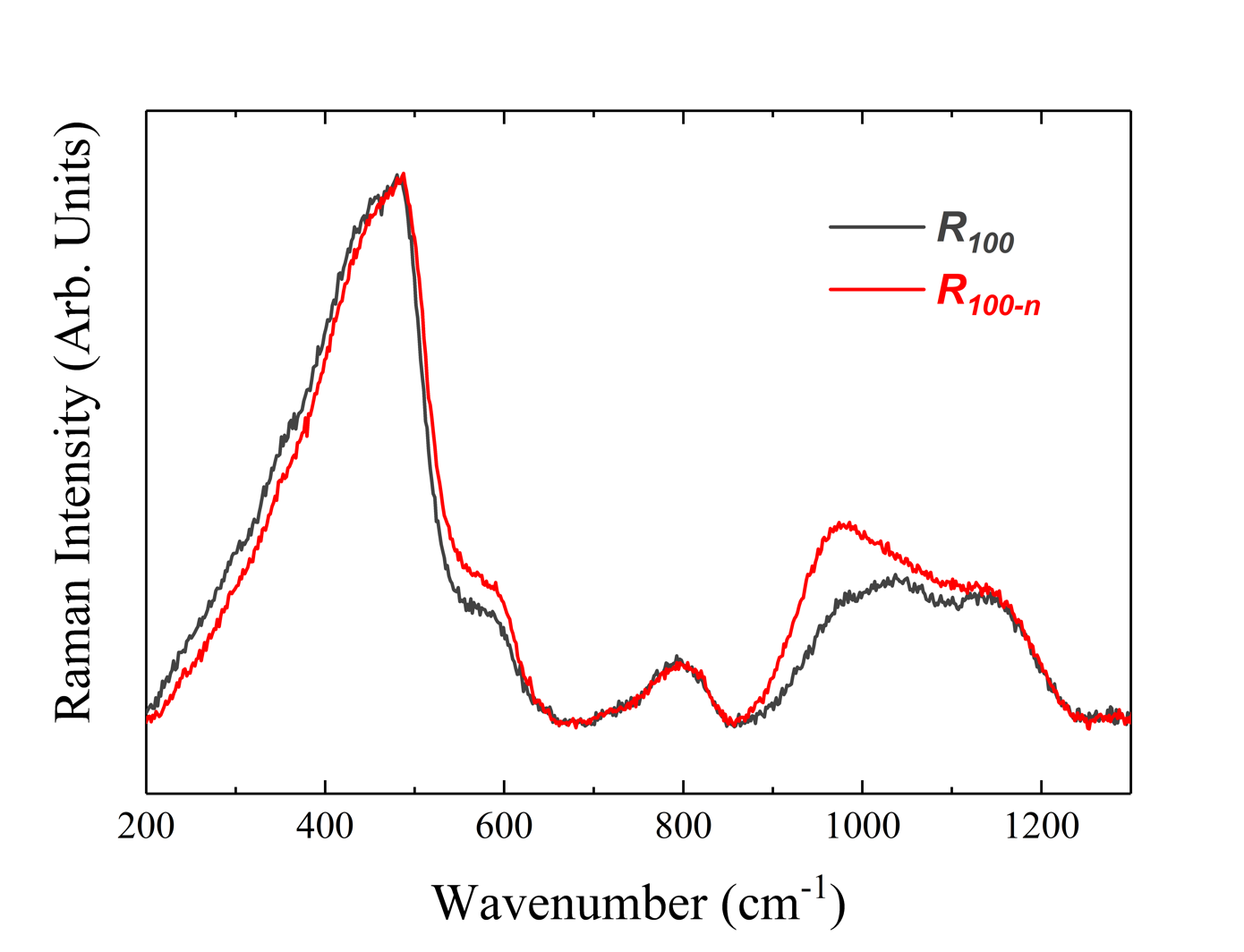


**Figure SI-1** Raman spectra of R_100_ (red) and R_100-n_ (grey). **The latter shows less** 5-, 6- fold tetrahedral (T) rings 250–500 cm^-1^; analogous 4-fold T rings (D_1_ band 500-550 cm^-1^); more 3-fold T rings (D_2_ band at 600 cm^-1^). ﻿Note the pronounced bump at 960 cm^-1^ ascribed to the Fe^3+^ asymmetric stretching vibrations.

***References***

[1] F. Vetere, G. Iezzi, H. Behrens, F. Holtz, G. Ventura, V. Misiti, A. Cavallo, S. Mollo, M. Dietrich, *Earth-Science Rev.* **2015**, *150*, 25.

[2] M. Cassetta, D. Di Genova, M. Zanatta, T. Boffa Ballaran, A. Kurnosov, M. Giarola, G. Mariotto, *Sci. Rep.* **2021**, *11*, 13072.

[3] D. Di Genova, D. Bondar, A. Zandonà, P. Valdivia, R. Al-Mukadam, H. Fei, A. C. Withers, T. Boffa Ballaran, A. Kurnosov, C. McCammon, J. Deubener, T. Katsura, *Chem. Geol.* **2023**, *625*.

[4] G. Giuliani, D. Di, F. Di, P. Valdivia, S. Mollo, C. Romano, T. Boffa, A. Kurnosov, A. Vona, *Chem. Geol.* **2024**, *670*, 122408.

[5] F. Vetere, S. Rossi, O. Namur, D. Morgavi, V. Misiti, P. Mancinelli, M. Petrelli, C. Pauselli, D. Perugini, *J. Geophys. Res. Planets* **2017**, *122*, 1522.

[6] A. Napolitano, E. G. Hawkins, *J. Res. Natl. Bur. Stand. Sect. A Phys. Chem.* **1964**, *68A*, 439.

[7] A. Sipp, D. R. Neuville, P. Richet, **1997**, *211*, 281.

[8] M. Cassetta, G. Mariotto, N. Daldosso, E. De Bona, M. Biesuz, G. D. Sorarù, R. Almeev, M. Zanatta, F. Vetere, *Minerals* **2023**, *13*, 1166.

[9] J. Schroeder, R. Mohr, *J. Am. Ceram. Soc.* **1973**, 510.

[10] D. Di Genova, A. Zandona, J. Deubener, *J. Non. Cryst. Solids* **2020**, *545*, 120248.

[11] D. Di Genova, D. Bondar, A. Zandonà, P. Valdivia, R. Al-Mukadam, H. Fei, A. C. Withers, T. Boffa Ballaran, A. Kurnosov, C. A. McCammon, J. Deubener, T. Katsura, *Chem. Geol.* **2023**, *625*.

[12] J. Schroeder, W. Wu, J. L. Apkarian, M. Lee, L. G. Hwa, C. T. Moynihan, *J. Non. Cryst. Solids* **2004**, *349*, 88.

[13] M. H. Manghnani, A. Hushur, T. Sekine, J. Wu, J. F. Stebbins, Q. Williams, *J. Appl. Phys.* **2011**, *109*.

[14] J. Schroeder, R. Mohr, I. ? B. Macedo, C. J. Montrose, *J. Am. Ceram. Soc.* **1973**, *56*, 510.

[15] J. P. Poole, *J. Am. Ceram. Soc.* **1949**, *32*, 230.

[16] J. O. Bockris, D. C. Lowe, *Proc. R. Soc.* **1954**, *226*, 423.

[17] A. G. Kalampounias, S. N. Yannopoulos, G. N. Papatheodorou, *J. Chem. Phys.* **2006**, *125*.
